# Genetic delimitation of *Pristimantisorestes* (Lynch, 1979) and *P.saturninoi* Brito et al., 2017 and description of two new terrestrial frogs from the *Pristimantisorestes* species group (Anura, Strabomantidae)

**DOI:** 10.3897/zookeys.864.35102

**Published:** 2019-07-18

**Authors:** Veronica L. Urgiles, Paul Székely, Diana Székely, Nicholas Christodoulides, Juan C. Sanchez-Nivicela, Anna E. Savage

**Affiliations:** 1 Department of Biology, University of Central Florida, 4110 Libra Drive, Orlando, Florida, 32816, USA; 2 Instituto Nacional de Biodiversidad del Ecuador INABIO, Rumipamba 341 y Av. De los Shyris, Quito, Ecuador; 3 Museo de Zoología, Universidad Técnica Particular de Loja, San Cayetano Alto, calle París s/n, 11-01-608, Loja, Ecuador; 4 EcoSs Lab, Departamento de Ciencias Biológicas, Universidad Técnica Particular de Loja, San Cayetano Alto, calle Marcelino Champagnat s/n, 11-01-608, Loja, Ecuador; 5 Faculty of Natural and Agricultural Sciences, Ovidius University Constanța, Constanța, Romania; 6 Laboratory of Fish and Amphibian Ethology, Behavioural Biology Unit, FOCUS, University of Liège, Liège, Belgium; 7 Grupo de Investigación Evolución y Ecología de Fauna Neotropical (EEFN), Universidad Nacional de Colombia, Bogotá D.C., Colombia; 8 Museo de Zoología, Universidad del Azuay, Cuenca, Ecuador

**Keywords:** Andes, Ecuador, new species, *P.cajanuma* sp. nov., *P.quintanai* sp. nov., Andes, Ecuador, nuevas especies, *P.cajanuma* sp. nov., *P.quintanai* sp. nov.

## Abstract

In the genus *Pristimantis*, species are often combined into taxonomic units called species groups. The taxonomy of these groups is frequently inaccurate due to the absence of genetic data from type series and repeated misidentifications generated by high morphological resemblance between taxa. Here, we focus on the *P.orestes* species group, providing the first genetic assessment of *P.orestes* sensu stricto from individuals collected from the type locality, with a reviewed diagnosis and description of advertisement calls. We find that two lineages previously named *P.orestes* are genetically distinct and should be separated into two different species. Based on genetic and morphological data, we name one of these species *P.cajanuma***sp. nov**. This new species is morphologically distinct from other members of the group by having shagreen dorsal skin, evident dorsolateral folds, broader discs on toes and fingers and pale gray ventral coloration. Additionally, *P.saturninoi* is placed within the *P.orestes* species group based on genetic data from its type series. However, we find that one of its paratypes is genetically distinct and belongs to a clade containing a new species we name *P.quintanai***sp. nov.** This new species is morphologically distinguished by lacking a tympanic membrane and vocal sacs in males, and by having expanded discs on toes and fingers, finely tuberculated dorsal skin and irregular white or cream spots in the groin and concealed surfaces of thighs. Our findings highlight the importance of providing genetic characterization and placement from the type series in taxonomic challenging groups, such as *Pristimantis*. We also suggest that the diversity of species within the *P.orestes* group will increase as more sampling is achieved in the southern Andes of Ecuador.

## Abstract (es)

Las especies pertenecientes al género *Pristimantis* usualmente están agrupadas en unidades taxonómicas llamadas grupos de especies. A menudo la taxonomía de estos grupos es problemática debido a la ausencia de información genética de las series tipo de las especies o debido a identificaciones erróneas generadas por la elevada similitud morfológica entre especies. Aquí nos enfocamos en el grupo de especies *P.orestes* y proveemos la primera evaluación genética de *P.orestes* sensu stricto en base a individuos colectados en la localidad tipo de la especie con una diagnosis revisada y descripción de vocalizaciones. Encontramos que dos linajes previamente nombrados como *P.orestes* son genéticamente distintos y deberían ser considerados como dos distintas especies. En base a evidencia genética y morfológica nombramos a una de estas especies *P.cajanuma***sp. nov.** La nueva especie es distinta de otras especies del grupo por presentar piel dorsal con textura finamente granular, pliegues dorsolaterales evidentes, discos amplios en dedos de pie y manos y una coloración ventral gris pálido. Adicionalmente, *P.saturninoi* es colocada dentro del grupo de especies *P.orestes* en base a información genética de especímenes tipo. Sin embargo, encontramos que uno de los paratipos es genéticamente distinto y está dentro de un clado que incluye a una nueva especie morfológicamente similar que nombramos como *P.quintanai***sp. nov.** Esta nueva especie se distingue de otros *Pristimantis* del grupo por carecer de una membrana timpánica diferenciada, machos sin sacos vocales y por presentar discos expandidos en los dedos de pies y manos, una piel dorsal con textura finamente tubercular y manchas irregulares blancas o crema-blanquecinas en las ingles y superficies ocultas de los muslos. Nuestros resultados resaltan la importancia de proveer caracterizaciones genéticas de especímenes tipos en grupos taxonómicamente complejos como los *Pristimantis*. Sugerimos que la diversidad de especies dentro del grupo de especies *P.orestes* incrementara a medida que más expediciones de campo se realicen en el sur de los Andes de Ecuador.

## Introduction

*Pristimantis* is a species-rich genus of terrestrial frogs that inhabit Central and South America (Hedges et al. 2008, Pinto-Sanchez et al. 2012). Although the genus is distributed broadly across this area, most of the diversity is restricted to the Andean regions of Ecuador, Colombia and Peru (Kieswetter and Schneider 2013). In Ecuador, 228 species of *Pristimantis* have been described to date, which remarkably represent over 40% of the known amphibians in the country (Ron et al. 2019).

Due to the extraordinary diversity and taxonomic complexity of the genus, *Pristimantis* species were grouped into phenetic taxonomic categories called species groups (Lynch and Duellman 1997). These groups were delimited based on a handful of morphological characteristics and resulted in the recognition of 11 species groups (Lynch and Duellman 1997). Such classifications are imperfect because they do not account for genetic and intraspecific variation or character plasticity within *Pristimantis*, yet they are useful in allowing us to recognize potentially diagnostic aspects of the morphology and natural history of individual species. The incorporation of molecular data in an increasing number of taxonomic analyses has recovered some species groups within *Pristimantis* as monophyletic, such as the *P.myersi* species group (Hedges et al. 2008). However, taxonomic resolution within most species groups remains unclear (Padial et al. 2014, Guayasamin et al. 2018), particularly in those groups where sufficient taxon sampling has not been accomplished and where molecular data from type series of described species (holotypes or paratypes) is not available.

Within this context, an interesting taxon that was recently recovered as a monophyletic clade using molecular phylogenetics is the *Pristimantisorestes* species group (Brito et al. 2017). When first proposed, the group included only three species from the south of Ecuador. As more samples were analyzed, a total of 14 species from southern Ecuador and northern Peru were suggested to be part of the group (Duellman and Lehr 2009). Only four of these species were included in the comprehensive Terrarana systematic revision proposed by Hedges et al. (2008) and in the later work of Padial et al. (2014). In both studies, the group was not recovered as monophyletic. Recently, Brito et al. (2017) provided a phylogenetic analysis of the group including a larger number of samples, and recovered monophyly but suggested that the *P.orestes* species group is restricted to the south of Ecuador and includes *P.andinognomus* (Lehr & Coloma, 2008), *P.bambu* (Arteaga & Guayasamin, 2011), *P.mazar* (Guayasamin & Arteaga, 2013), *P.muranunka* (Brito et al., 2017), *P.orestes* (Lynch, 1979) and *P.simonbolivari* (Wiens & Coloma, 1992). In contrast, the Peruvian species *P.melanogaster* (Duellman & Pramuk, 1999) and *P.simonsii* (Boulenger, 1900), which were previously placed in the *P.orestes* group by Lynch and Duellman (1997), are members of different clades. Similar results were found by Székely et al. (2018), with the inclusion of the newly described species *P.tiktik* (Székely et al., 2018) within the *P.orestes* group.

While the analyses of Brito et al. (2017) and Székely et al. (2018) have increased our knowledge of the phylogenetic relationships in the *P.orestes* group, these molecular analyses have also identified polytomies generated by erroneous assignment of species to multiple different clades due to morphological misidentifications and because good quality DNA samples are unavailable from formalin-fixed type specimens (most notably, *P.orestes*). Thus, further genetic characterizations are essential to accurately delimit species within the *P.orestes* species group. Molecular systematics is also necessary to accurately place those taxa that were suggested to be part of the *P.orestes* group but were described using morphological data only, such as *P.saturninoi* (Brito et al., 2017). Here, we present a novel exploration of the nuclear and mitochondrial molecular diversification of the *P.orestes* species group using broad spatial sampling across high elevation ecosystems in the southern Andes of Ecuador. Specifically, we provide (1) a redescription and genetic delimitation of *P.orestes* sensu stricto, representing the first genetic assessment of the species from its type locality, (2) a revised placement of *P.saturninoi*, and (3) a description of two new species that are part of the *P.orestes* species group.

## Materials and methods

Amphibians were collected under authorization from the Ecuadorian Environmental Ministry (MAE): MAE-DNB-CM-2015-0016, MAE-DNB-CM-2016-0045 and MAE-DPC-AIC-B-2018-003. All animal research was carried out under the University of Central Florida’s IACUC protocol #18-16W and approved by the Ethics Committee of Universidad Técnica Particular de Loja (UTPL-CBEA-2016-001). Specimens were euthanized with a solution of 2% lidocaine following McDiarmid et al. (1994), fixed in 10% formalin, and preserved in 70% ethanol. Tissue samples from liver were extracted and preserved in 96% ethanol. Geographic coordinates and elevation were recorded with a GPS unit (WGS84 datum). Descriptions of the habitat where specimens were collected and coloration patterns in life are based on the authors’ field notes and photographs. Individuals collected in the province of Cañar were deposited at the Museo de Zoología de la Universidad del Azuay (MZUA), Ecuador, whereas individuals collected in the Loja Province were deposited in the Museo de Zoología, Universidad Técnica Particular de Loja (MUTPL), Ecuador.

Because we aimed to provide a genetic delimitation of *P.orestes* sensu stricto, we collected specimens from the type locality of *P.orestes* described in Lynch (1979) at 11 km NE Urdaneta, Loja Province in Ecuador. We reviewed the morphological characteristics of the collected specimens with the original descriptions and with the type specimens (holotypes and paratypes) available in the Kansas Museum of Natural History (KU). We also included individuals collected in Cajanuma, Loja Province, that were previously identified as *P.orestes* in earlier phylogenetic analyses. Finally, we included samples from the type series of *P.saturninoi* as well as from individuals from three nearby localities in the province of Cañar that shared similar morphological characteristics and where previously identified as *P.saturninoi*.

### DNA extraction, amplification and sequencing

Total DNA was extracted from liver tissue using DNeasy Blood & Tissue kits (Qiagen, Valencia, California, USA) following the manufacturer’s protocol. We amplified two mitochondrial genes (12S and 16S) and one nuclear gene (RAG-1). We obtained a 658 bp fragment of 12S using forward primer 12L29 (5’-AAAGCRTAGCACTGAAAATGCTAAGA-3’) and reverse primer 12H46 (5’-GCTGCACYTTGACCTGACGT-3’) (Heinicke et al. 2007). To obtain a 1080 bp fragment for 16S, we aligned the fragment obtained with forward primer 16L19 (5’-AATACCTAACGAACTTAGCGATAGCTGGTT-3’) and reverse primer 16H36 (5’-AAGCTCCAWAGGGTCTTCTCGTC-3’) (Heinicke et al. 2007), and the fragment obtained with forward primer 16SC (5’-GTRGGCCTAAAAGCAGCCAC-3’) and reverse primer 16Sbr-H (5’-CCGGTCTGAACTCAGATCACGT-3’) (Darst and Cannatella 2004, Palumbi et al. 1991). We also obtained a 654 bp fragment of RAG-1 using forward primer R182 (5’-GCCATAACTGCTGGAGCATYAT-3’) and reverse primer R270 (5’-AGYAGATGTTGCCTGGGTCTTC-3’) (Heinicke et al. 2007). PCR conditions follow those specified by Heinicke et al. (2007) for 12S, RAG-1, and the 16S fragment obtained with primers 16L19 and 16H36. For the 16S fragment obtained with primers 16SC and 16SBR, we used PCR conditions specified in Guayasamin et al. (2017). For the samples we could not amplify under these conditions, the annealing temperature was lowered to 49 °C. The final volume of each PCR reaction was 20 μL and contained 2 μL of 10mM dNTP, 3.6 μL of OneTaq PCR buffer, 2 μL of each primer (10 μM) and 0.3 μL of 1 U OneTaq Polymerase and 1μM of DNA. PCR amplification products were cleaned using ExoSAP PCR Product Cleanup Reagent (Thermo Fisher scientific) and Sanger sequenced in both directions by Eurofins Genomics (Kentucky, USA).

### Phylogenetic analysis and genetic distances

In addition to newly generated sequence data, we conducted BLAST searches to identify similar sequences of 12S, 16S and RAG-1 in GenBank. The searches show most similarity with the *Pristimantisorestes* species group: *P.simonbolivari* (identity 95%, accession number: EF493671), *P.mazar* (identity 96%, accession number KY967664), *P.orestes* (identity 99%, accession number EF493388), *P.tiktik* (identity 94%, accession number MH668274). Therefore, we included all available sequences of the *Pristimantisorestes* species group available in GenBank. To correctly place the *P.orestes* group within the broader *Pristimantis* phylogeny, we included sequences from close congeneric clades based on the phylogeny proposed by Padial et al. (2014) and defined *Strabomantisbiporcatus* and *Lynchiusflavomaculatus* as outgroups. A summary of GenBank accession numbers, museum collection identifiers and localities are given in Table 1.

**Table 1.** Species of *Pristimantis* included in this analysis. For each specimen, we provide the museum number, source, locality and GenBank accession number. (*) indicates the outgroup taxa. Museum abbreviations are as follows: MZUA (Museo de Zoología-Universidad del Azuay, Ecuador), MUTPL (Museo de Zoología, Universidad Técnica Particular de Loja, Ecuador), MEPN (Museo de Historia Natural de la Escuela Politecnica Nacional, Ecuador), KU (Kansas Museum of Natural History, USA), QCAZ (Museo de Zoología-Pontificia Universidad Católica del Ecuador, Ecuador), DHMECN (Departamento de Herpetologia, Instituto Nacional de Biodiversidad del Ecuador, Ecuador).

**Species**	**Museum number**	**GenBank accession number**			**Locality**
		***12S***	***16S***	***RAG–1***	
* Pristimantis andinognomus *	QCAZ45661	–	KY967671	KY967690	Ecuador: Zamora Chinchipe, Tapichalaca Reserve
	QCAZ45534	–	KY967669	KY967688	Ecuador: Loja, Podocarpus National Park, guardianía Cajanuma
* P. bambu *	QCAZ46744	–	KY967659	KY967693	Ecuador: Cañar, Reserva Mazar
	QCAZ46708	–	KY967673	–	Ecuador: Cañar, Reserva Mazar
* P. cajanuma *	MUTPL160	MK993333	MK604537	–	Ecuador: Loja, Cajanuma, Podocarpus National Park, Los Miradores Trail
	MUTPL157	MK993331	MK604535	–	Ecuador: Loja, Cajanuma, Podocarpus National Park, Los Miradores Trail
	MUTPL158	MK993332	MK604536	MK602184	Ecuador: Loja, Cajanuma, Podocarpus National Park, Los Miradores Trail
* P. ceuthospilus *	KU212216	EF493520	EF493520	–	Peru: Cajamarca, Chota, 12 km W Llama
* P. chalceus *	KU177638	EF493675	EF493675	–	Ecuador: Carchi, Maldonado
* P. cryophilius *	KU217863	EF493672	EF493672	–	Ecuador: Azuay, 4 km W Laguna Torcadorn
* P. diadematus *	KU221999	EU186668	EU186668	–	Peru: Loreto, Teniente Lopez
* P. galdi *	QCAZ32368	EU186670	EU186670	EU186746	Ecuador: Zamora Chinchipe, El Pangui
* P. imitatrix *	KU215476	EF493824	EF493667	–	Peru: Madre de Dios, Cuzco Amazonico, 15 km E Puerto Maldonado
* P. mazar *	QCAZ27559	–	KY967664	KY967683	Ecuador: Cañar, Reserva Mazar, La Libertad
	QCAZ27572	JF906315	KY967666	KY967685	Ecuador: Cañar, Reserva Mazar, La Libertad
* P. melanogaster *	MHNSM56846	EF493826	EF493664	–	Peru: Amazonas, N. Slobe Abra Barro Negro, 28 km SSW Leimebambe
* P. muranunka *	MEPN14737	–	KY967661	KY967680	Ecuador: Zamora Chinchipe, Cerro Plateado
	MEPN14722	–	KY967660	KY967679	Ecuador: Zamora Chinchipe, Cerro Plateado
* P. orestes *	KU218257	EF493388	EF493388	–	Ecuador: Azuay, 7 km E Sigsig
	QCAZ45464	JF906323	–	–	Ecuador: Loja, Podocarpus National Park, guardianía Cajanuma
	QCAZ45646	JF906324	–	–	Ecuador: Loja, Podocarpus National Park, guardianía Cajanuma
	MUTPL242		MK604538	MK602185	Ecuador, Loja, 11 km NE Urdaneta
	MUTPL248	MK993330	MK604539	MK602186	Ecuador, Loja, 11 km NE Urdaneta
	MUTPL249		MK604540	–	Ecuador, Loja, 11 km NE Urdaneta
	MZUA.AN.2488		MK604545	MK602190	Ecuador, Loja, 11 km NE Urdaneta
	QCAZ45556	–	KY967670	KY967689	Ecuador: Loja, Podocarpus National Park, Lagunas del Compadre
* P. parvillus *	KU177821	EF493352	EF493352	–	Ecuador: Pichincha
* P. phoxocephalus *	KU218025	EF493349	EF493349	–	Ecuador: Chimborazo, 70 km W Riobamba via Pallatanga
* P. quintanai *	MZUA.AN.1748	–	MK604542	MK602187	Ecuador: Cañar, Rivera
	MZUA.AN.1881	MK993335	MK604541	MK602188	Ecuador: Cañar, Comunidad Guangras
	MZUA.AN.1878	MK993334	MK604543	–	Ecuador: Cañar, Guangras
	MZUA.AN.2705	MK993337	MK604546	MK602191	Ecuador: Cañar, Llavircay
	MZUA.AN.1900	MK993336	MK604544	MK602189	Ecuador: Cañar, Llavircay
* P. rhodoplichus *	KU219788	EF493674	EF493674	–	Peru: Piura, Le Tambo
* P. saturninoi *	DHMECN 12237	MK993329	MK604534	–	Ecuador: Morona–Santiago, Sangay National Park
	DHMECN 12232	MK993327	MK604533	–	Ecuador: Morona–Santiago, Sangay National Park
	DHMECN 12214	MK993328	MK604532	–	Ecuador: Morona–Santiago, Sangay National Park
* P. simonbolivari *	QCAZ56567		KY967676	KY967695	Ecuador: Bolívar, Bosque Protector Cashca Totoras
	KU218254	EF493671	EF493671	–	Ecuador: Bolívar, Bosque Protector Cashca Totoras
* P. simonsii *	KU212350	EU186665	EU186665	–	Peru: Cajamarca, S slope Abra Quilsh, 28 km NNW Cajamarca
*Pristimantis* sp.	QCAZ56535	–	KY967675	KY967694	Ecuador: Azuay, Laguna Patococha
*Pristimantis* sp.	DHMECN3112	–	KY967658	KY967677	Ecuador: Zamora Chinchipe, Reserva Tapichalaca
* P. spinosus *	KU218052	EF493673	EF493673	–	Ecuador: Morona–Santiago, 10.6 km W Plan de Milogio
* P. tiktik *	MUTPL239	MH668274	MH668275	MH708575	Ecuador: Loja, 21 km E Urdaneta
	MUTPL247	MH668161	MH668276	MH708576	Ecuador: Loja, 14 km E Urdaneta
* P. unistrigatus *	KU218057	EF493387	EF493387	EF493444	Ecuador: Imbabura, 35 km E Pquela
* Lynchius flavomaculatus *	KU218210*	EU186667	EU186667	EU186745	Ecuador: Morona–Santiago, Yangana
* Strabomantis biporcatus *	CVULA7073*	EU186691	EU186691	EU186754	Venezuela: Sucre, Parque Nacional de Paria, Les Melenas, Peninsula de Paria

Sequences were cleaned, assembled and aligned in GeneiousPro v. 9.1.6 (Biomatters Ltd.) using the MAFFT algorithm (Katoh and Standley 2013). Manual posterior corrections of the alignment were performed to remove unnecessary gaps and to adjust the correct reading frame in the RAG-1 gene alignment. To detect possible alignment errors and significant incongruences, we first constructed single-gene trees. A Maximum Likelihood (ML) phylogenetic analysis was performed in IQ-TREE (Nguyen et al. 2015) with each individual gene alignment. Nodal support was obtained after generating 1000 samples for ultrafast bootstrap. Next, we concatenated 12S, 16S and RAG-1 alignments into a single matrix to conduct phylogenetic analyses based on all genes. We used PartitionFinder2 (Lanfear et al. 2016) under the corrected Bayesian information criterion to find the best model of evolution. Molecular phylogenetic relationships with the concatenated matrix were inferred using ML and Bayesian inference. ML analysis were conducted in IQ-TREE in the CIPRES Science Gateway portal (Miller et al. 2010). Nodal support was obtained after generating 1000 samples for ultrafast bootstrap. Bayesian inference was conducted in MrBayes v.3.1.2 (Ronquist et al. 2012) under Markov chain Monte Carlo sampling. We performed two independent runs of 50,000,000 generations and four chains sampling every 100 generations. The first 100,000 generations were discarded as burn-in. To visualize the generated samples from the Bayesian analysis and confirm that the posterior probability had reached a stationary local maximum, we used TRACER (Rambaut et al. 2014). The average standard deviation of split frequencies was < 0.05 and effective sample size was > 2000 for all parameters. We compared genetic distance between clades and between each individual sequence using uncorrected p distances for the 16S fragment in MEGA X (Kumar et al. 2018).

### Morphological analysis

The format of the description follows Lynch and Duellman (1997) and the format of the diagnostic characters follows Duellman and Lehr (2009). Sex of each specimen was determined via direct observation of secondary sexual traits (vocal slits and vocal sac) and gonadal inspection through abdominal incisions. Morphometric variables are based on Watters et al. (2016). We measured each variable three times using a digital caliper to the nearest 0.1 mm. We present the average, maximal, and minimal values of each morphometric character. Abbreviations for measurements are as follows: eye to nostril distance (EN), head length (HL), head width (HW), interorbital distance (IOD), internarial distance (IND), snout vent length (SVL), tibia length (TL), foot length (FL), tympanum diameter (TD), eye diameter (ED) and upper eyelid width (EW).

For the species comparison we reviewed morphological characteristics, measurements and coloration patterns of morphologically similar members of the *P.orestes* species group (Lynch and Duellman 1997) and additional similar terrestrial frogs that occur in southern Ecuador: *P.andinognomus*, *P.bambu*, *P.mazar*, *P.orestes*, *P.vidua* (Lynch, 1979), *P.simonbolivari*, *P.tiktik* and *P.saturninoi*. We based our comparisons on original descriptions of the species and via direct examination of type material available in the Kansas Museum of Natural History (KU, USA), the Zoology Museum of the Catholic University of Ecuador (QCAZ, ECU), Zoology Museum of Azuay University (MZUA, ECU), and the Instituto Nacional de Biodiversidad del Ecuador (DHMECN, ECU). Reviewed specimens are listed in Suppl. material 1.

### Call recording

The calls of four *P.orestes* sensu stricto males were recorded in the field in August 2016 using an Olympus LS-11 Linear PCM Recorder and a RØDE NTG2 condenser shotgun microphone at 44.1 kHz sampling frequency and 16-bit resolution, in WAV file format (Suppl. material 2). Air temperature and humidity were measured with a data logger (Lascar Electronics, model EL-USB-2-LCD, accuracy: ± 0.5 °C; ± 5%). The original, analyzed call recordings are deposited in full length in the Fonoteca UTPL (Suppl. material 3). Acoustic analysis was conducted using Raven Pro 1.4 (http://www.birds.cornell.edu/ raven). We measured the temporal parameters from the oscillograms and the spectral parameters from spectrograms obtained through Hanning window function, DFT: 512 samples, 3 dB filter bandwidth: 124 Hz, 50% overlap and 86.1 Hz frequency resolution.

The terminology and procedures for measuring call parameters follow Cocroft and Ryan (1995), Toledo et al. (2015) and Köhler et al. (2017) and a call-centered approach was used to distinguish between a call and a note (sensu Köhler et al. 2017). The following temporal and spectral parameters were measured and analyzed: (1) call duration: time from the beginning to the end of a call; (2) inter-call interval: the interval between two consecutive calls, measured from the end of one call to the beginning of the consecutive call; (3) call rate: number of calls per second, measured as the time between the beginning of the first call and the beginning of the last call; (4) dominant frequency: the frequency containing the highest sound energy, measured along the entire call; and (5) the 90% bandwidth, reported as frequency 5% and frequency 95%, or the minimum and maximum frequencies, excluding the 5% below and above the total energy in the selected call.

## Results

### Molecular systematics

**Figure 1.**
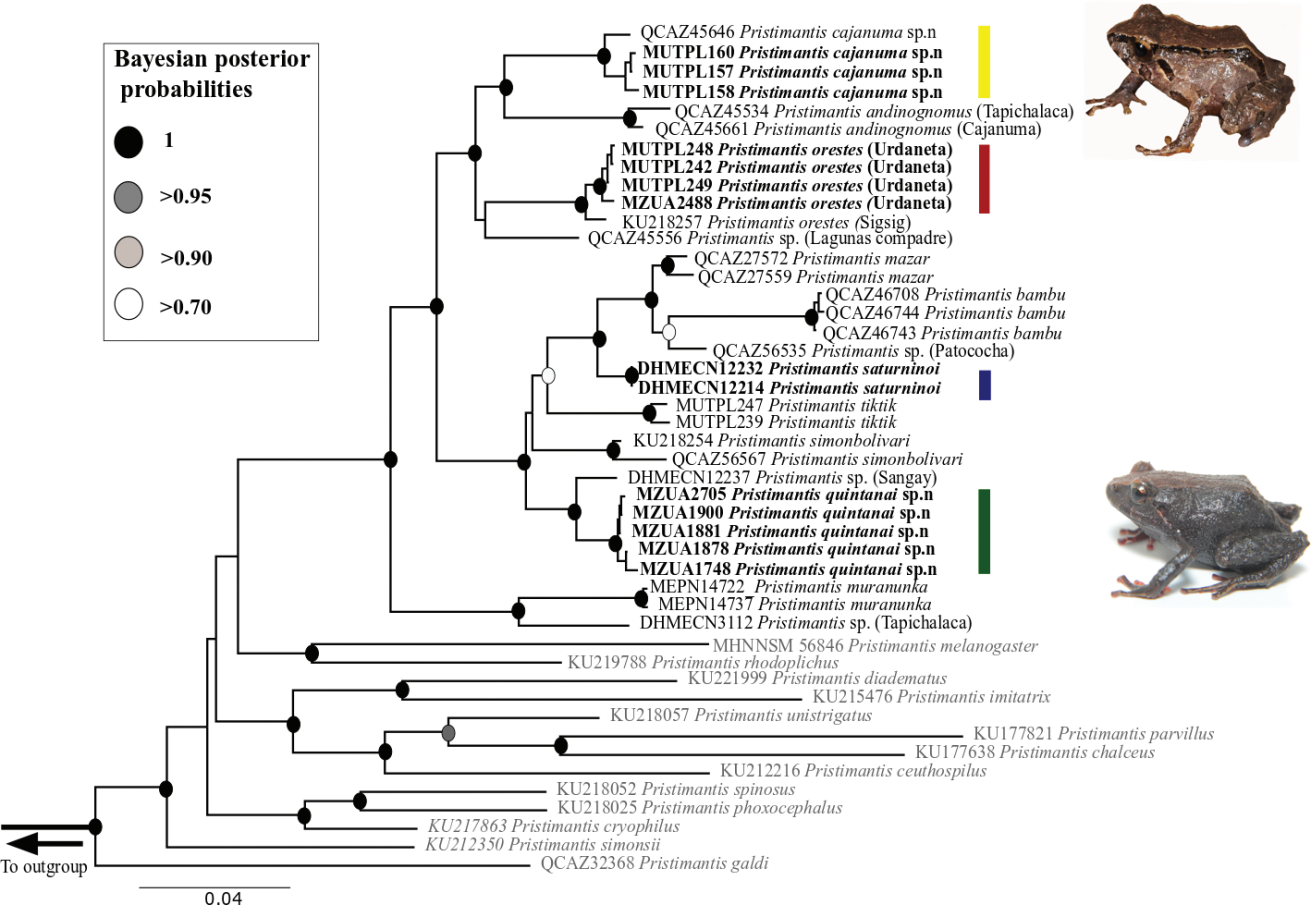
Bayesian phylogeny of the *Pristimantisorestes* species group based on 2393 base pairs of concatenated DNA from 12S, 16S, and RAG-1 gene fragments. Bayesian posterior probability support values are shown for each node, except when they are less than 0.70. Bolded names represent new sequences obtained in this study. Colored bars represent the following species: *P.cajanuma* sp. nov. (yellow), *P.orestes* (dark red), *P.saturninoi* (blue) and *P.quintanai* sp. nov. (green). We rooted the tree with *Lynchiusflavomaculatus* and *Strabomantisbiporcatus*. Names in gray represent closely related clades of the *Pristimantisorestes* species group based on the phylogeny of Padial et al. (2014). Photographs of the new species *P.cajanuma* and *P.quintanai* are shown.

Phylogenetic analyses were based on newly generated sequences from 16 individuals. The final dataset (46 terminals) including the three concatenated gene fragments consisted of 2393 bp, including 658 bp of 12S, 1080 bp of 16S and 654 bp of RAG-1. We recovered some minor differences between the RAG-1 single-gene tree and our concatenated tree, but only for very poorly supported nodes (Suppl. material 4). The best partition scheme included four subsets. The first partition subset included the 12S sequences and the best substitution model was GTR+G, and the second partition subset included 16S sequences and the best substitution model was GTR+I+G. The subset for RAG-1 was partitioned according to codon positions. Subset three included RAG-1 1^st^ and 2^nd^ codon positions and the best substitution model was GTR+G. Subset four included RAG-1 3^rd^ codon positions and the best substitution model was HKY+I. We found no conflict between topologies recovered with Bayesian inference and those inferred with ML (Suppl. material 5).We recovered the *P.orestes* species group as monophyletic with strong support (bootstrap values [bb] = 100%; posterior probabilities [pp] = 100) in both ML and Bayesian analysis (Fig. 1). Our analysis also recovered *P.orestes* sensu stricto as a well-supported clade (bb = 100%; pp = 1) that includes four individuals from the type locality, Urdaneta (MUTPL 248, MUTPL 242, MUTPL 249, MZUA.AN.2488) and one individual from Sigsig (KU 18257). The *P.orestes* sensu stricto clade is the sister species of an as-yet undescribed *Pristimantis* from Lagunas del Compadre (QCAZ 45556). *Pristimantissaturninoi* and the two new species, *P.cajanuma* sp. nov. and *P.quintanai* sp. nov., were recovered within the *P.orestes* species group. *Pristimantiscajanuma* sp. nov. is the sister species of *P.andinognomus*, and *P.quintanai* sp. nov. is the sister species of the clade containing *P.simonbolivari*, *P.tiktik*, and the clade that contains *P.mazar*, *P.bambu*, two undescribed species of *Pristimantis*, and *P.saturninoi* (DHMECN 12232, DHMECN 12214). However, one of the paratypes of *P.saturninoi* (DHMECN 12237) is genetically distinct from the other *P.saturninoi* specimens DHMECN 12232 and DHMECN 12214 (genetic distance = 6%; Table 1) and is placed as the sister species of *P.quintanai* sp. nov. (genetic distance to *P.quintanai* = 2.3%).

The genetic distance between *P.cajanuma* and its sister species *P.andinognomus* is 6% and the genetic distance between *P.orestes* (Urdaneta + Sigsig) and the specimen QCAZ 45556 from Lagunas del Compadre is 7% (Table 2). The genetic distances between *P.saturninoi* (the clade including individuals DHMECN 12232 and DHMECN 123214) and *P.mazar* and *P.bambu* are 3% and 6%, respectively (Table 2).

**Table 2.** Genetic uncorrected pairwise distances (%) among clades of the *Pristimantisorestes* species group. Numbers at the top of the table correspond to the first column. The number of individuals for each comparison is shown above the diagonal. Bold numbers correspond to intraspecific genetic distances.

	1	2	3	4	5	6	7	8	9	10	11	12	13	14
1. *P.tiktik*	**(0.01)**	*N* = 5	*N* = 4	*N* = 3	*N* = 7	*N* = 7	*N* = 4	*N* = 4	*N* = 5	*N* = 4	*N* = 3	*N* = 2	*N* = 4	*N* = 3
2. *P.cajanuma* sp. nov.	0.06	**(0.0)**	*N* = 5	*N* = 4	*N* = 8	*N* = 8	*N* = 5	*N* = 5	*N* = 6	*N* = 5	*N* = 4	*N* = 4	*N* = 5	*N* = 4
3. *P.saturninoi*	0.05	0.06	**(0.0)**	*N* = 3	*N* = 7	*N* = 7	*N* = 4	*N* = 4	*N* = 5	*N* = 4	*N* = 3	*N* = 3	*N* = 4	*N* = 3
4. *P.* sp. (Sangay)	0.06	0.06	0.06	–	*N* = 6	*N* = 6	*N* = 3	*N* = 3	*N* = 4	*N* = 3	*N* = 2	*N* = 2	*N* = 3	*N* = 2
5. *P.quintanai* sp. nov.	0.05	0.07	0.05	0.02	**(0.0)**	*N* = 10	*N* = 7	*N* = 7	*N* = 8	*N* = 7	*N* = 6	*N* = 6	*N* = 7	*N* = 6
6. *P.orestes*	0.07	0.06	0.07	0.07	0.07	**(0.01)**	*N* = 7	*N* = 7	*N* = 8	*N* = 7	*N* = 6	*N* = 6	*N* = 7	*N* = 6
7. *P.simonbolivari*	0.05	0.06	0.05	0.05	0.05	0.07	**(0.02)**	*N* = 4	*N* = 5	*N* = 4	*N* = 3	*N* = 3	*N* = 4	*N* = 3
8. *P.andinognomus*	0.07	0.06	0.07	0.08	0.08	0.07	0.07	**(0.02)**	*N* = 5	*N* = 4	*N* = 3	*N* = 3	*N* = 4	*N* = 3
9. *P.bambu*	0.06	0.08	0.06	0.07	0.08	0.09	0.07	0.08	**(0.0)**	*N* = 5	*N* = 4	*N* = 4	*N* = 5	*N* = 4
10. *P.mazar*	0.06	0.07	0.03	0.06	0.06	0.08	0.05	0.07	0.05	**(0.01)**	*N* = 3	*N* = 3	*N* = 4	*N* = 3
11. *P.* sp. (Patococha)	0.05	0.06	0.04	0.07	0.07	0.08	0.05	0.07	0.05	0.02	–	*N* = 2	*N* = 3	*N* = 2
12. *P.* sp. (Lagunas del Compadre)	0.05	0.05	0.07	0.07	0.07	0.07	0.05	0.06	0.06	0.05	0.06	–	*N* = 2	*N* = 2
13. *P.muranunka*	0.08	0.09	0.10	0.10	0.11	0.10	0.09	0.10	0.09	0.09	0.09	0.10	**(0.0)**	*N* = 3
14. *P.* sp. (Tapichalaca)	0.08	0.09	0.10	0.09	0.10	0.10	0.08	0.09	0.09	0.09	0.09	0.09	0.07	–

### Taxonomic treatment

#### Class Amphibia Linnaeus, 1758

##### Order Anura Fischer von Waldheim, 1813

###### Superfamily Brachycephaloidea Günther, 1858

####### Family Strabomantidae Hedges, Duellman & Heinicke, 2008

######## Subfamily Pristimantinae Pyron & Wiens, 2011

######### Genus *Pristimantis* Jiménez de la Espada, 1870

########## Pristimantis orestes

Taxon classificationAnimaliaAnuraStrabomantidae

(Lynch, 1979)

bfd714b7-29e8-4532-8bd1-0c04a95e0cfd

Fig. 2

Eleutherodactylus orestes Lynch, 1979
Eleutherodactylus (Eleutherodactylus) orestes : Lynch and Duellman 1997
Pristimantis orestes : Heinicke et al. 2007
Pristimantis (Pristimantis) orestes : Hedges et al. 2008

########### Etymology.

Greek, Orestes, a mountaineer.

**Type material. Holotype.**KU141998, an adult female, obtained 11 km NE Urdaneta, Provincia Loja, Ecuador, 2970 m, 24 July 1971 by William E. Duellman and Bruce MacBryde.

**Paratypes**. KU141999–KU142003, collected syntopically with the holotype.

########### Diagnosis.

*Pristimantisorestes* is a small species distinguished by the following combination of traits: (1) skin on dorsum finely tuberculated (in life the skin tuberculated texture is more evident); evident dorsolateral folds absent but sometimes a continuous row of pustules is present; low middorsal fold present; skin on venter areolate; discoidal fold weak, more evident posteriorly; (2) tympanic membrane absent but tympanic annulus evident, its length about 45% of the length of eye; supratympanic fold present; (3) snout short, subacuminate in dorsal view, rounded in profile; canthus rostralis weakly concave in dorsal view, rounded in profile; (4) upper eyelid bearing several small tubercles, similar in size and shape with the ones from the dorsum, about 90% IOD in females and 60% IOD in males; cranial crests absent; (5) dentigerous processes of vomers prominent, oblique, slightly ovoid, separated medially by distance lower than width of processes; each processes bearing 3 to 6 teeth; (6) males with a subgular vocal sac and small vocal slits; nuptial pads absent; (7) Finger I shorter than Finger II; discs on fingers just slightly expanded, rounded; circumferential grooves present; (8) fingers lacking lateral fringes; subarticular tubercles prominent; supernumerary palmar tubercles present, smaller than subarticular tubercles; palmar tubercle completely divided into a larger (inner) and a smaller (outer) tubercles; thenar tubercle oval, smaller than the inner palmar tubercle; (9) small, inconspicuous, ulnar tubercles present (trait more visible in life); (10) heel with small tubercles; outer edge of tarsus with a row of small tubercles; inner tarsal tubercles coalesced into a short tarsal fold (traits more visible in life); (11) inner metatarsal tubercle broadly ovoid, about 2× ovoid, subconical (in profile), outer metatarsal tubercle; supernumerary plantar tubercles present; (12) toes lacking lateral fringes; webbing basal; Toe V slightly longer than Toe III; discs on toes just slightly expanded, rounded, about same size as those on fingers; circumferential grooves present; (13) in life, dorsum varies from gray, copper-brown and brown; venter gray to pale brown spotted with cream and/or brown; groin, anterior and posterior surfaces of thigh, concealed shank and axillae are dark brown or black enclosing large white spots; iris whitish gray, with a reddish broad median horizontal streak, and with fine black reticulations; (14) SVL 22.4–23.7 mm in adult females (*N* = 2) and 16.5–22.3 mm in adult males (20.1 ± 2.16 SD, *N* = 5).

########### Variation.

**Figure 2.**
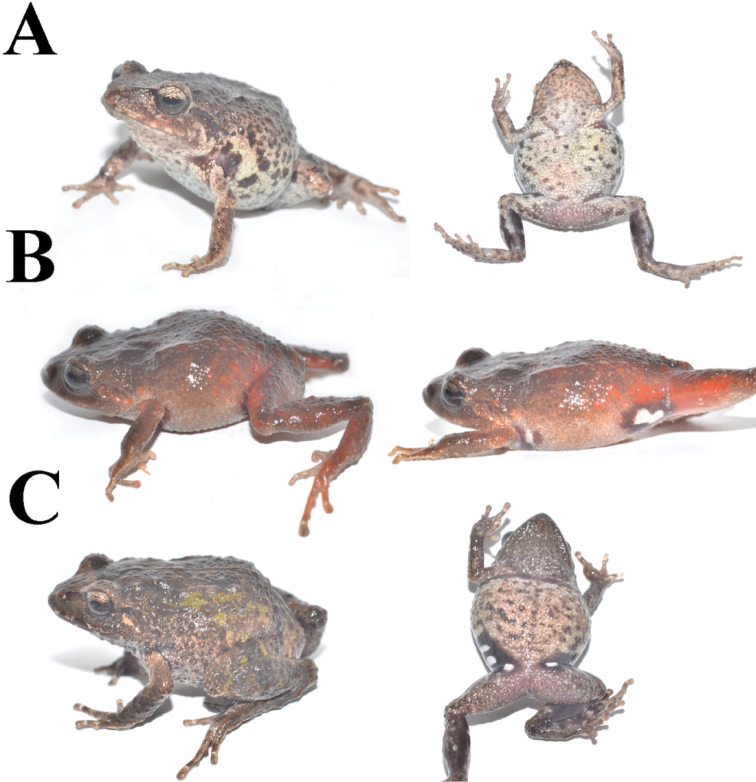
*Pristimantisorestes* variation in life. **A** MZUA.AN.2488, profile and ventral view **B** MZUA.AN.2493, profile view **C** MZUA.AN.2497, profile and ventral view.

Morphometric variation is shown in Table 3. In one male (MZUA.AN.2497) the discoidal fold is more visible but the ulnar tubercles are barely distinguishable both in life and in preservative. In one female (MZUA.AN.2493) a vague dorsolateral fold (formed by a continuous row of pustules) is present in the anterior half of dorsum and the middorsal fold is not distinguishable; in this same individual the ulnar tubercles are more notorious than in the other preserved specimens. In a female (MZUA.AN.2497), the dorsum and dorsal surfaces of limbs display a brownish-green coloration; the flanks and lips are dark brown with irregular cream blotches. One male (MZUA.AN.2488) presents orange spots over a brown background in the dorsum, flanks and limbs and a continuous orange blotch in the groin and anterior surfaces of thighs (Fig. 2).

**Table 3.** Measurements (in mm) of adult males and females of *Pristimantisorestes* collected from Urdaneta. The mean and standard deviation (SD) of each morphological character are shown for males (*N* = 5) but not females due to sample size (*N* = 1). Abbreviations of the morphometric measurements are presented in Materials and methods.

	MZUA 2488 ♀	MZUA 2493 ♂	MZUA 2497 ♂	MUTPL 242 ♂	MUTPL 248 ♂	MUTPL 249 ♂	Mean ± SD (range) ♂
**SVL**	22.4	20.0	16.5	20.7	20.8	22.3	20.1 ± 2.2 (16.5–22.3)
**EN**	2.2	1.7	1.5	1.7	1.7	1.8	1.7 ± 0.1 (1.5–1.8)
**TD**	1.5	0.9	0.8	1.2	1.3	1.4	1.1 ± 0.3 (0.7–1.4)
**ED**	2.5	2.3	2.0	2.4	2.4	2.5	2.3 ± 0.2 (2.0–2.5)
**EW**	1.8	1.8	1.4	1.6	1.8	2.0	1.7 ± 0.2 (1.4–2.0)
**IOD**	3.1	2.4	2.2	2.9	2.9	3.1	2.7 ± 0.4 (2.2–3.1)
**IND**	1.8	1.8	1.4	1.9	1.6	2.1	1.8 ± 0.3 (1.4–2.1)
**HL**	6.7	5.5	6.1	7.4	7.4	7.6	6.8 ± 0.9 (5.5–7.6)
**HW**	6.5	8.3	6.8	7.7	7.5	7.9	7.6 ± 0.6 (6.8–8.3)
**TL**	9.2	9.0	8.2	9.0	9.0	9.4	8.9 ± 0.4 (8.2–9.4)
**FL**	8.2	8.3	7.8	8.7	8.7	8.9	8.5 ± 0.4 (7.8–8.9)

########### Advertisement call.

**Figure 3.**
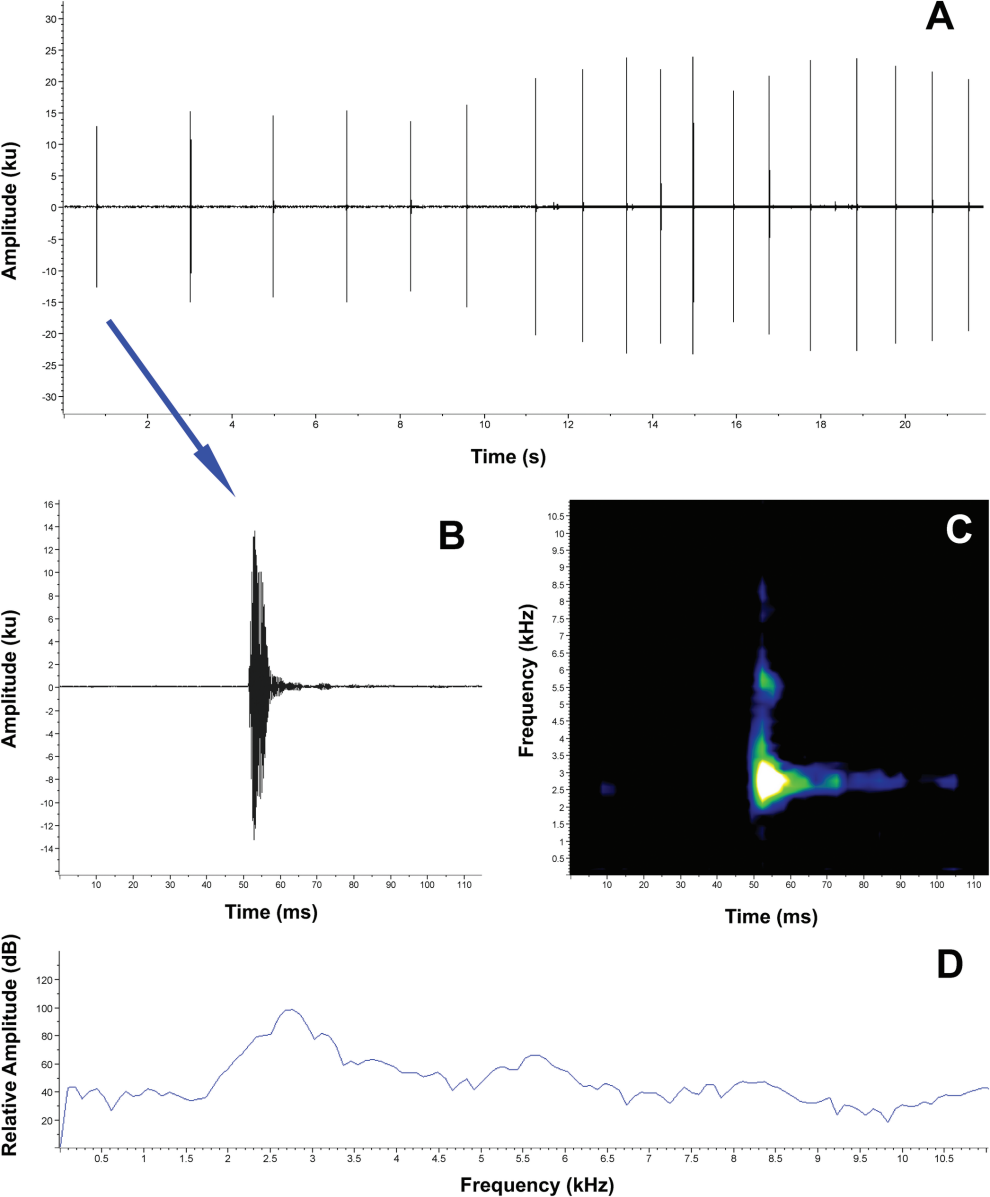
Advertisement call of *Pristimantisorestes*. **A** Oscillogram of a 12-call section of the call series **B** Oscillogram of a single call **C** Spectogram of a single call **D** Power spectrum of a single call.

Two of the analyzed recordings (FUTPL-A-130 and FUTPL-A-131) are from the same unvouchered male. *Pristimantisorestes* has an advertisement call characterized by a call series composed by clicking calls repeated for long periods of time (Fig. 3). Because the males can call continuously for long periods of time, the call series duration is unknown. The calls are characterized by a duration of (range and mean ± SD in parenthesis): 0.008–0.013 s (0.011 ± 0.0009, *N* = 190), an inter-call interval of 0.705–3.824 s (1.680 ± 0.650, *N* = 185) and a call rate of 0.50–0.73 calls/s (0.58 ± 0.110, *N* = 4). The 90% bandwidth ranged from 2325.6–2756.2 Hz (2605.7 ± 88.555, *N* = 190) to 2756.2–3186.9 Hz (2989.2 ± 115.100, *N* = 190), with the dominant frequency being at 2670.1–2928.5 Hz (2773.5 ± 77.359, *N* = 190). The fundamental frequency is not recognizable, but 2 to 3 harmonics are sometimes visible. Three of the four recorded males increased the call rate at the end of their calls (Fig. 3), intensifying the call emissions in the last 20–30 seconds. The call rate increased, and the inter-call interval decreased from 0.35–0.63 calls/s (0.47 ± 0.145, *N* = 3) to 0.70–1.06 calls/s (0.88 ± 0.177, *N* = 3), respectively, and from 1.063–3.824 s (2.111 ± 0.672, *N* = 64) to 0.705–2.087 s (1.253 ± 0.401, *N* = 88).

########### Distribution.

**Figure 4.**
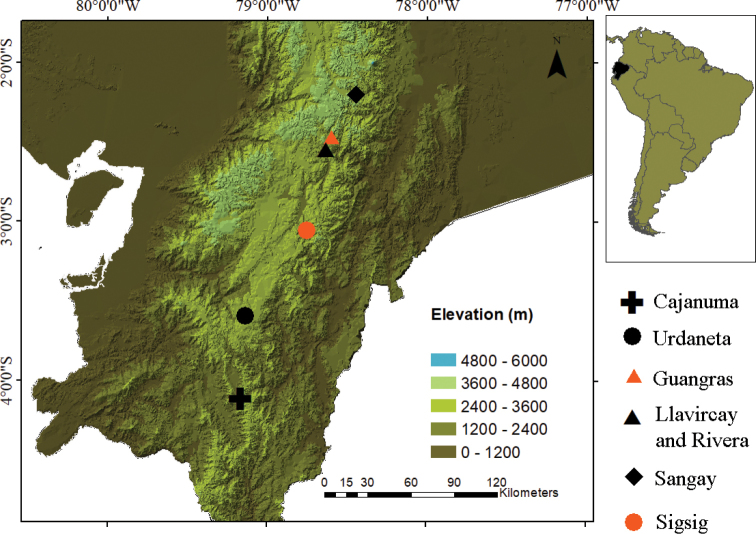
Map of southern Ecuador showing recording localities of *Pristimantissaturninoi, P.quintanai* sp. nov., *P.orestes*, and *P.cajanuma* sp. nov.

Lynch (1979) states that this species occurs on the eastern Andean Cordillera from the Cuenca hoya to the Loja hoya in southern Ecuador. However, we suggest that this distribution might be inaccurate and needs to be reviewed, as many of the records are probably erroneous belonging to very similar, but in fact different species. For example, additional localities previously reported by Lynch (1979) from the Loja Province include Saraguro, but this record is likely erroneous, and refers to observations of an undescribed, very similar species. Guayasamin and Arteaga (2013) also reported *P.orestes* from Susudel in the Azuay province (MZUTI 706), but this record needs to be reviewed via molecular and morphological analysis to confirm identity of this specimens. Thus, we recommend limiting the distribution of *P.orestes* to the confirmed localities in Urdaneta and in Sigsig, in an elevational range between 2940 to 3100 m (Fig. 4).

########### Natural history.

We found all the specimens in a pastureland in a subpáramo habitat. Specimens were encountered at night on grassy vegetation (usually at 10–20 cm above the ground) near the road. Calling males were encountered between May and August. The only sympatric frog species registered was *Gastrothecapseustes*.

########### Conservation status.

*Pristimantisorestes* is categorized as endangered based on criteria B1b(iii) (IUCN 2018). We suggest maintaining this category because the species i) has only been found in two localities, and ii) its natural habitat (páramo and subpáramo) has been heavily damaged and fragmented by grazing, fires and roads. Also, in its type locality, *P.orestes* is not locally abundant, only few individuals were registered at every visit to the population. However, additional information is needed to evaluate population trends and to assess the presence and impact of pathogenic infections in this species.

########### Remarks.

Lynch (1979) provides an accurate and detailed description of this species, including a brief description of the cranial osteology. Our diagnosis concurs with all the morphological features described by the author, but we also focus on characters that were not detailed in the original description but that are useful to distinguish *P.orestes* from other similar species (e.g., condition of discoidal fold and nuptial pads in males). The only significant difference is that the outer tarsal tubercles are not prominent, and we consider the color of the iris to be whitish gray instead of gray-bronze (Fig. 2). The diagnosis provided herein is based on four specimens from the original description (KU 141998, 141999, 142000, 142002): one adult female (MZUA.AN.2488) and five adult males (MUTPL 242, 248, 249 and MZUA.AN.2493, 2497) collected from the type locality.

########## Pristimantis cajanuma sp. nov.

Taxon classificationAnimaliaAnuraStrabomantidae

3fc70173-97d4-4838-8a9f-017c72c473f0

http://zoobank.org/B00AB277-06B5-4F73-84E1-38EBC5E870D8

Figs 5 , 6 , 7 , 8

########### Type material.

**Figure 5.**
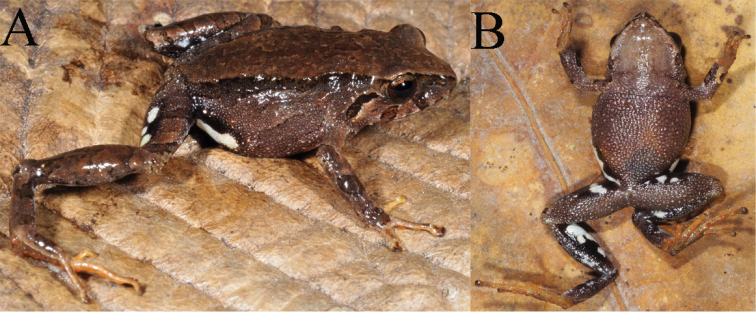
Holotype of *Pristimantiscajanuma* sp. nov. in life. **A** dorsolateral view **B** ventral view.

**Figure 6.**
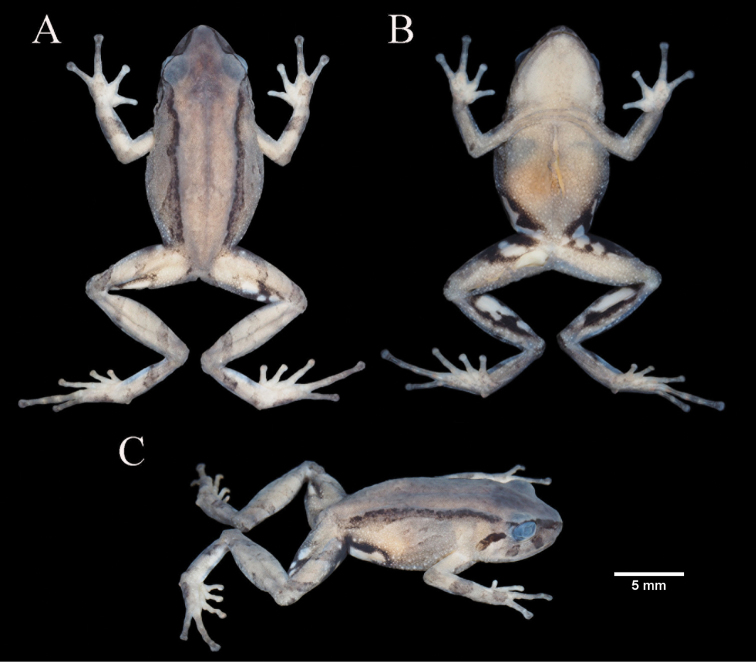
Holotype of *Pristimantiscajanuma* sp. nov. in preservative, adult female MUTPL 346: **A** dorsal view **B** ventral view **C** lateral view.

**Figure 7.**
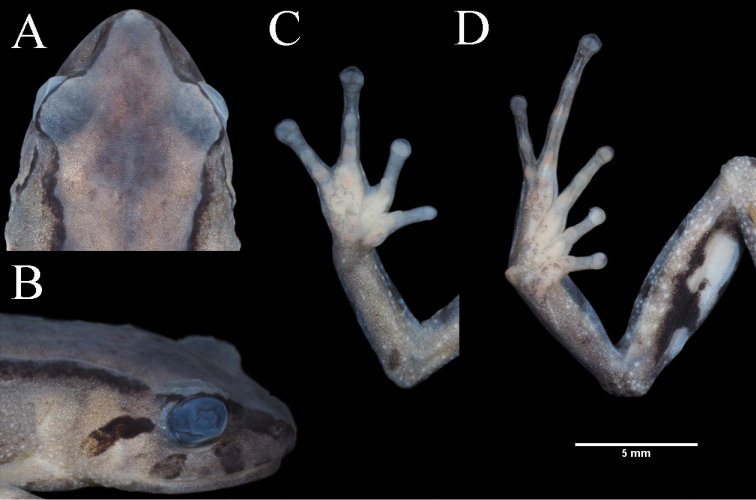
Holotype of *Pristimantiscajanuma* sp. nov. in preservative, adult female MUTPL 346: **A** dorsal view of head **B** profile view of head **C** palmar surfaces **D** plantar surfaces.

**Holotype**. MUTPL 346 (Figs 5–7), field no. SC 159, adult female from Ecuador, Loja Province, Loja canton, Cajanuma entrance to the Podocarpus National Park, on Los Miradores trail (4.1176S, 79.1663W; datum WGS84), 3022 m above sea level, collected by Diana Székely and Paul Székely on 28 June 2018.

**Figure 8.**
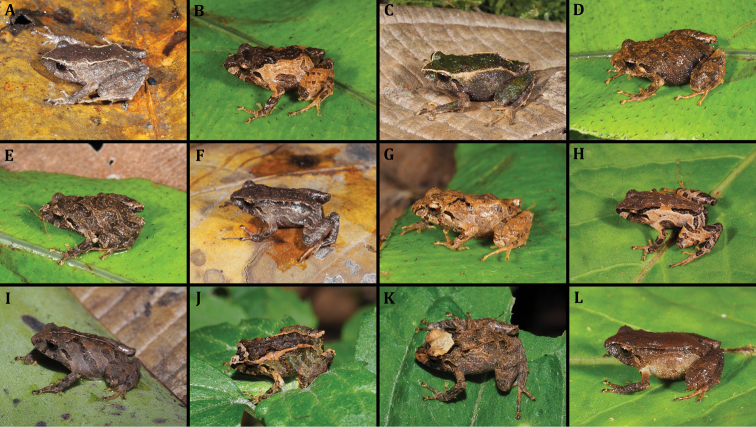
Color variation of *Pristimantiscajanuma* sp. nov. in life. **A–E** females: **A** MUTPL 343 **B** MUTPL 591 **C** MUTPL 347 **D** MUTPL 583 **E** MUTPL 584; **F–H** juveniles: **F** MUTPL 345 **G** MUTPL 588 **H** MUTPL 592 **I–L** males: **I** MUTPL 353 **J** MUTPL 352 **K** MUTPL 355 **L** MUTPL 594.

**Paratypes** (Fig. 8) 16 specimens collected in the type locality: MUTPL 343 (SC 156) an adult female and MUTPL 344 (SC 157) a juvenile (4.1170S, 79.1668W; datum WGS84), 2974 m, MUTPL 345 (SC 158) a juvenile (4.1176S, 79.1663W; datum WGS84), 3022 m, MUTPL 347 (SC 160) an adult female and MUTPL 353 (SC 166) a subadult male (4.1177S, 79.1658W; datum WGS84), 3042 m, and MUTPL 352 (SC 165) a subadult male and MUTPL 355 (SC 168) an adult male (4.1177S, 79.1647W; datum WGS84), 3098 m collected by Diana Székely and Paul Székely on 28 June 2018; MUTPL 573 (SC 331) a subadult female (4.1166S, 79.1691W; datum WGS84), 2890 m collected by Diana Székely and Paul Székely on 09 December 2018; MUTPL 583 (SC 903) an adult female and MUTPL 584 (SC 904) a subadult female (4.1167S, 79.1690W; datum WGS84), 2883, MUTPL 588 (SC 908) and MUTPL 592 (SC 912) two juveniles, MUTPL 589 (SC 909) and MUTPL 591 (SC 911) two subadult females, and MUTPL 593 (SC 913) and MUTPL 594 (SC 914) two adult males (4.1169S, 79.1666W; datum WGS84), 2984 m collected by Diana Székely and Paul Székely on 05 January 2019.

########### Diagnosis.

*Pristimantiscajanuma* is a small species distinguished by the following combination of traits: (1) skin on dorsum shagreen; skin on venter areolate (trait more visible in life); discoidal fold weak; dorsolateral folds present; low middorsal fold present; (2) tympanic membrane absent but tympanic annulus evident, its length about 45% of the length of eye; supratympanic fold present; (3) snout short, subacuminate in dorsal view, rounded in profile; canthus rostralis concave in dorsal view, angular in profile; (4) upper eyelid bearing several small tubercles, about 60% IOD in females and 65% IOD in males; cranial crests absent; (5) dentigerous processes of vomers prominent, triangular, without space between the processes; each processes bearing 4 to 7 teeth; (6) males with subgular vocal sac and vocal slits; nuptial pads absent; (7) Finger I shorter than Finger II; discs on fingers expanded, rounded; circumferential grooves present; (8) fingers lacking lateral fringes; subarticular tubercles prominent; supernumerary palmar tubercles present, rounded, smaller than subarticular tubercles; palmar tubercle bifurcated (partially divided distally); thenar tubercle oval; (9) small, inconspicuous, ulnar tubercles present (trait more visible in life); (10) heel with small tubercles; outer edge of tarsus with a row of small tubercles; inner tarsal tubercles coalesced into a short tarsal fold; (11) inner metatarsal tubercle broadly ovoid, about 2× round, subconical (in profile) outer metatarsal tubercle; supernumerary plantar tubercles present; (12) toes lacking lateral fringes; webbing basal; Toe V slightly longer than Toe III; discs on toes expanded, rounded, about same size as those on fingers; circumferential grooves present; (13) in life, dorsum of various shades of brown, gray or sometimes green, with or without darker bands or bars; flanks various shades of brown or gray, usually lighter than the dorsum coloration; venter light gray with or without dark flecks; groin, anterior and posterior surfaces of thighs, concealed shanks and axillae are black enclosing large white spots; iris bronze with a reddish broad median horizontal streak, and with fine black reticulations; SVL 17.6–22.1 mm in adult females (19.8 ± 1.81 SD, *N* = 8) and 14.4–16.4 mm in adult males (15.4 ± 0.83 SD, *N* = 5).

########### Comparison with similar species.

**Figure 9.**
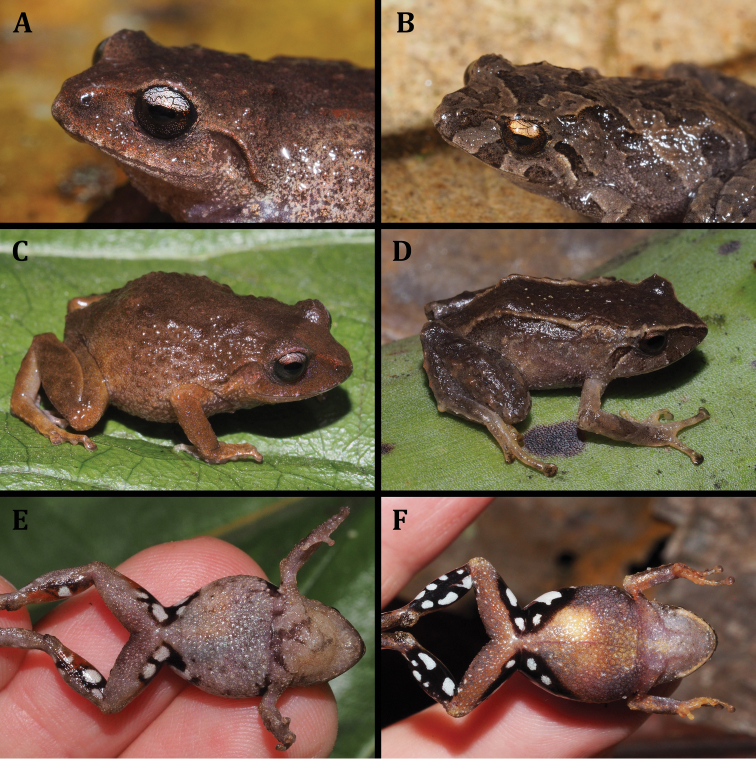
Morphological differences between *Pristimantisorestes* sensu stricto (A, C, E) and *P.cajanuma* sp. nov. (B, D, F): whitish gray iris (**A**) vs. bronze iris (**B**) dorsolateral folds absent (**C**) vs. dorsolateral folds present (**D**); and limited black coloration in the groin and concealed surfaces of shanks (**E**) vs. widespread black coloration (**F**).

*Pristimantiscajanuma* is morphologically similar to its closest relatives, the species from the recently redefined *P.orestes* group (sensu Brito et al. 2017), but its characteristic morphological features readily distinguish it from all resembling species. *Pristimantiscajanuma* is most similar to *P.orestes* sensu stricto but can be easily distinguished by having evident dorsolateral folds (absent in *P.orestes*), a shagreen skin on dorsum (finely tuberculated in *P.orestes*), broader discs on the fingers and toes (e.g. width of disc on Finger III in *P.cajanuma*: 0.8–1 mm, *N* = 3; in *P.orestes*: 0.6–0.7 mm, *N* = 3), palmar tubercle bifurcated, only partially divided distally (completely divided into a larger and a smaller tubercle in *P.orestes*) and by the more widespread black coloration in the groin and concealed shanks (Fig. 9). Its sister species, *P.andinognomus* is significantly smaller (females up to 17 mm, males up to 14 mm; Lehr and Coloma 2008), has the Toe V much longer than Toe III (Toe V slightly longer than Toe III in *P.cajanuma*) and lacks the typical black enclosing large white spots coloration of the groin, anterior and posterior surfaces of thighs, concealed shanks and axillae of *P.cajanuma*.

*Pristimantissimonbolivari* has a similar coloration of the groin, anterior and posterior surfaces of thighs, concealed shanks and axillae but lacks dorsolateral folds (present in *P.cajanuma*) and its venter coloration is darker, orange or brown (light gray in *P.cajanuma*). *Pristimantissaturninoi* and *P.quintanai* sp. nov. also have similar coloration of the groin, thighs and shanks but *P.saturninoi* has a black or blackish-amber venter (venter light gray in *P.cajanuma*) and green iris (bronze in *P.cajanuma*). *Pristimantisquintanai* sp. nov. is different by having a finely tubercular dorsum skin (shagreen in *P.cajanuma*), and by having a black, reddish-brown or reddish-cream venter coloration.

All other species of the *P.orestes* group (sensu Brito et al. 2017) lack the typical coloration of the groin, thighs, shanks and axillae of *P.cajanuma*: *Pristimantisbambu* has large yellow spots; *P.mazar* has a reticulated pattern, *P.tiktik* presents a black reticulum in the females and whitish/pinkish yellow coloration in the males and *P.muranunka* shows a brown or dark brown uniform coloration.

########### Description of holotype.

Adult female (MUTPL 346) (Figs 5–7) with head narrower than body, wider than long, HL 92% of HW, HW 36% of SVL; HL 33% of SVL; snout short (snout to eye distance 16% of SVL), subacuminate in dorsal view, rounded in profile (Fig. 7A, B); canthus rostralis concave in dorsal view, angular in profile; loreal region flat; ED notably greater than eye-nostril distance; nostrils not protuberant; lips not flared; cranial crests absent; upper eyelid bearing several small tubercles (one slightly larger than the others), width of upper eyelid 64% of IOD; half of tympanic annulus evident (Fig. 7B), oval (slightly higher than wider), its upper and posterodorsal part obscured by rounded supratympanic fold; tympanic membrane absent; diameter of tympanum 52% of the length of eye; postrictal tubercles are fused and form a short ridge situated posteroventrally to tympanic annulus; choanae small, round, partially concealed by palatal shelf of maxillary arch; dentigerous processes of vomers prominent, triangular in outline, much larger than the choanae, without space between the processes, each bearing 4 or 5 teeth; tongue 1.5× as long as wide, slightly notched posteriorly, posterior half not adherent to floor of mouth.

Skin on dorsum shagreen, that on flanks is finely tuberculated; thin, low middorsal fold starting at tip of snout and ending at cloaca; long, continuous dorsolateral folds present (Fig. 6A); skin of throat shagreen, that on chest and belly areolate; discoidal fold weak, barely visible (Fig. 6B); ornamentation in cloacal region absent.

Ulnar tubercles small, inconspicuous (trait more visible in life); outer palmar tubercle inconspicuous, bifurcated (partially divided distally); thenar tubercle oval; subarticular tubercles prominent, round and subconical in section; supernumerary palmar tubercles rounded, smaller than subarticular tubercles; fingers lacking lateral fringes; Finger I shorter than Finger II; discs on fingers expanded, rounded; all fingers bearing pads well defined by circumferential grooves (Fig. 7C).

Hindlimbs short; TL 50% of SVL; FL 47% of SVL; heel with small tubercles (one slightly larger than the others); outer edge of tarsus with a row of small tubercles (trait more visible in life); inner edge of tarsus bearing a short fold; inner metatarsal tubercle broadly ovoid, about 2× round and subconical (in profile) outer metatarsal tubercle; subarticular tubercles prominent, round and subconical in section; plantar supernumerary tubercles rounded, smaller than subarticular tubercles; toes lacking lateral fringes; webbing basal; discs on toes expanded, rounded, about same size as those on fingers; toes with ventral pads well defined by circumferential grooves; relative length of toes I <II < III < V < IV; Toe V slightly longer than Toe III (tip of Toe III not reaching the penultimate subarticular tubercle on Toe IV, tip of Toe V not reaching the proximal edge of distal subarticular tubercle on Toe IV) (Fig. 7D).

########### Measurements of holotype.

SVL 20.6; HW 7.5; HL 6.9; IOD 2.4; internarial distance 1.7; upper EW 1.5; ED 2.3; eye-nostril distance 1.8; snout to eye distance 3.2; TD 1.2; TL 10.2; FL 9.7.

Body mass of holotype: 1.01 g.

########### Coloration of holotype.

In life (Fig. 5) the dorsum is brown with dark mottling and with the dorsolateral folds blackish-dark brown. Flanks grayish-brown with white flecks. Dorsal surfaces of hindlimbs and arms the same color as the dorsum but with dark brown transverse bars. The head bears blackish-dark brown canthal, labial and supratympanic stripes. The throat is whitish gray and the venter is brownish-gray with white flecks. Groin, anterior and posterior surfaces of thighs, concealed shanks and axillae are black enclosing large white spots. The dorsal and ventral surfaces of the hands and feet are reddish-orange. The iris is bronze with a reddish broad median, horizontal streak, and with fine black reticulations.

In preservative (Figs 6, 7) the dorsum is brownish gray and the flanks whitish gray with white flecks. All the blackish-dark brown coloration of the dorsolateral folds, canthal, labial and supratympanic stripes in life became dark gray in preservative. Also, the black enclosing the large white spots of the groin, anterior and posterior surfaces of thighs, concealed shanks and axillae in life turned to dark gray in preservative. The dorsal and ventral surfaces of the hands and feet are whitish gray.

########### Variation.

Morphometric variation is shown in Table 4. The dorsolateral folds were fragmented in some of the specimens (Figs 8E, G, I, 9B) and thus not so evident, but all encountered individuals (probably more than 50) had dorsolateral folds. *Pristimantiscajanuma* displays a considerable variation in the dorsal coloration (Figure 8). We encountered individuals with a general gray (Fig. 8A, E, F, I, K), light brown (Fig. 8G), dark brown (Fig. 8D, L), light brown with a dark brown middorsal band (Fig. 8B, H) and even green (Fig. 8C, J) coloration. Some of the individuals had chevrons on the dorsum (Fig. 8E) and/or dark transverse bars on the flanks and limbs (Fig. 8E, I, J), white or yellowish dorsolateral folds (Fig. 8A, C, J), white middorsal fold (Fig. 8I) and even completely whitish-yellow head (Fig. 8K). As for the sexual dimorphism, besides the size difference (the males are significantly smaller), the only identified coloration difference is that the males are lacking the characteristic large white spots enclosed by black of the groin, anterior and posterior surfaces of thighs, concealed shanks and axillae. From the encountered individuals, only the specimen MUTPL 353 had a similar coloration of the groin, but significantly fainter.

**Table 4.** Measurements (in mm) of adult males and females of *Pristimantiscajanuma* sp. nov. Mean and standard deviation (SD) values of each morphological character are shown for females (*N* = 8) and males (*N* = 5). Abbreviations of the morphometric measurements are presented in Materials and methods.

	MUTPL													Mean ± SD (range) ♀	Mean ± SD (range) ♂
	343 ♀	346 ♀	347 ♀	573 ♀	583 ♀	584 ♀	589 ♀	591 ♀	352 ♂	353 ♂	355 ♂	593 ♂	594 ♂		
**SVL**	20.7	20.6	22.0	18.5	22.1	18.4	18.1	17.6	14.4	14.9	16.1	16.4	15.2	19.8 ± 1.8 (17.6–22.1)	15.4 ± 0.8 (14.4–16.4)
**EN**	1.7	1.8	1.7	1.7	1.7	1.6	1.6	1.6	1.3	1.3	1.3	1.3	1.3	1.7 ± 0.1 (1.6–1.8)	1.3 (1.3)
**TD**	1.1	1.2	1.2	0.9	1.2	0.8	0.9	0.9	0.7	0.9	0.9	0.8	0.7	1.1 ± 0.7 (0.8–1.2)	0.8 ± 0.1 (0.7–0.9)
**ED**	2.3	2.3	2.4	2.3	2.5	2.2	2.2	2.2	1.9	2.0	2.1	1.9	1.7	2.3 ± 0.1 (2.2–2.5)	1.9 ± 0.1 (1.7–2.1)
**EW**	1.5	1.5	1.6	1.2	1.7	1.4	1.2	1.3	1.2	1.2	1.3	1.2	1.2	1.4 ± 0.2 (1.2–1.7)	1.2 ± 0.1 (1.2–1.3)
**IOD**	2.5	2.4	2.5	2.4	2.5	2.2	2.4	2.1	1.7	1.8	2.0	2.0	1.9	2.4 ± 0.2 (2.1–2.5)	1.9 ± 0.1 (1.7–2.0)
**IND**	1.8	1.7	1.9	1.7	2.0	1.7	1.7	1.7	1.4	1.5	1.6	1.6	1.6	1.8 ± 0.1 (1.7–2.0)	1.5 ± 0.1 (1.4–1.6)
**HL**	6.8	6.9	7.6	5.8	7.5	5.8	5.8	5.7	5.2	5.4	5.7	5.7	5.3	6.5 ± 0.8 (5.7–7.6)	5.5 ± 0.2 (5.2–5.7)
**HW**	7.5	7.5	8.1	6.5	7.8	6.9	6.7	6.3	4.9	5.2	5.6	5.9	5.7	7.2 ± 0.7 (6.3–8.1)	5.5 ± 0.4 (4.9–5.9)
**TL**	10.5	10.2	10.7	9.5	10.8	9.3	9.2	9.1	7.3	7.7	8.0	8.0	7.9	9.9 ± 0.7 (9.1–10.8)	7.8 ± 0.3 (7.3–8.0)
**FL**	9.1	9.0	9.6	8.9	10.2	8.9	8.4	8.2	6.8	7.4	7.6	7.5	7.4	9.1 ± 0.6 (8.2–10.2)	7.3 ± 0.3 (6.8–7.6)

The dorsolateral folds are already visible in the juveniles (Fig. 8F, G, H), but the large white spots enclosed by black in the groin, anterior and posterior surfaces of thighs, concealed shanks and axillae are not so conspicuous and probably become darker and more evident as the animals mature. The identity of all the specimens was confirmed molecularly using the 16S mitochondrial gene.

########### Etymology.

The specific epithet *cajanuma* (in Quechua language “*cajan*” means cold and “*uma*” peak, or head, in other words the cold peak, referring to the cold climate of the area) is used as a noun in apposition and refers to the region where the species is found. Cajanuma is the highest entrance to the Podocarpus National Park, which is one of the largest and most diverse protected area from Ecuador. By naming this species *cajanuma* we also want to honor and recognize the Podocarpus National Park rangers for their extraordinary and tireless work protecting this incredible reserve.

########### Distribution and natural history.

*Pristimantiscajanuma* is known only from the Cajanuma entrance to the Podocarpus National Park, in an altitudinal range between 2882 and 3097 m a.s.l. in a Mountain Cloud Forest ecosystem. All specimens were encountered during the night, perching on the vegetation (usually at 10–40 cm above the ground), near the Los Miradores trail. No calling males were encountered. Other sympatric frog species include *Pristimantisandinognomus*, *P.vidua* and an undescribed species of *Pristimantis*.

########### Conservation status.

Even though *Pristimantiscajanuma* is currently known only from the type locality in the Podocarpus National Park, we recommend that this species to be categorized as Near Threatened following the IUCN criteria. This is due the fact that the species is locally abundant and its habitat does not face any major threats (because it is situated within a national protected area). However, at present its distribution is limited to only one locality, therefore there is some level of threat.

########## Pristimantis quintanai sp. nov.

Taxon classificationAnimaliaAnuraStrabomantidae

e064e445-9412-4677-974a-aff00d50488e

http://zoobank.org/33697F57-D0C2-470F-8750-5ECF80546904

Figs 10 , 11 , 12

########### Type material.

**Figure 10.**
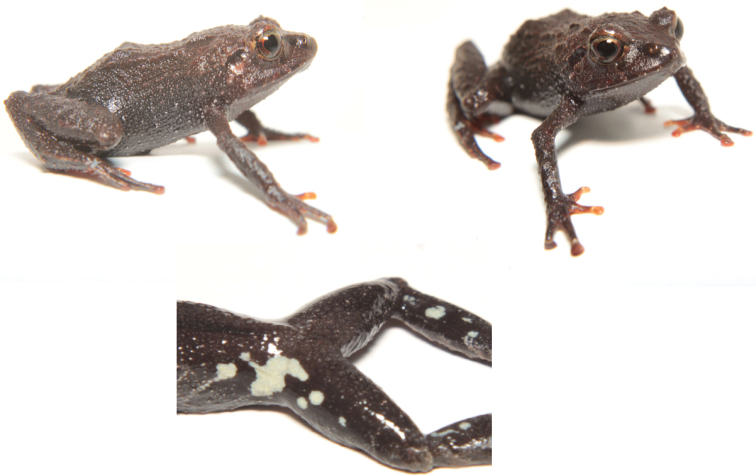
Holotype of *Pristimantisquintanai* sp. nov. in life.

**Figure 11.**
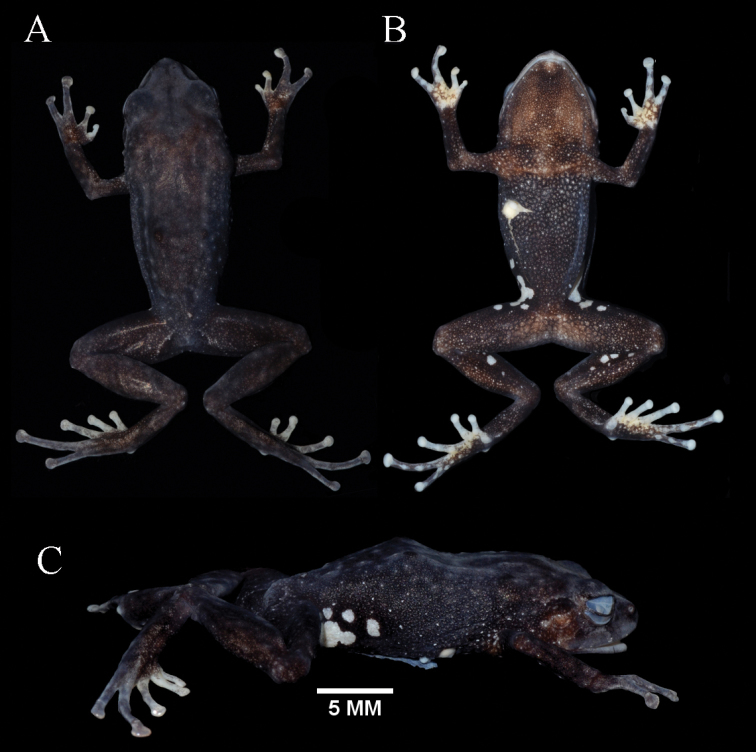
Holotype of *Pristimantisquintanai* sp. nov. in preservative, adult female MZUA.AN.1881. **A** dorsal view **B** ventral view **C** profile view.

**Figure 12.**
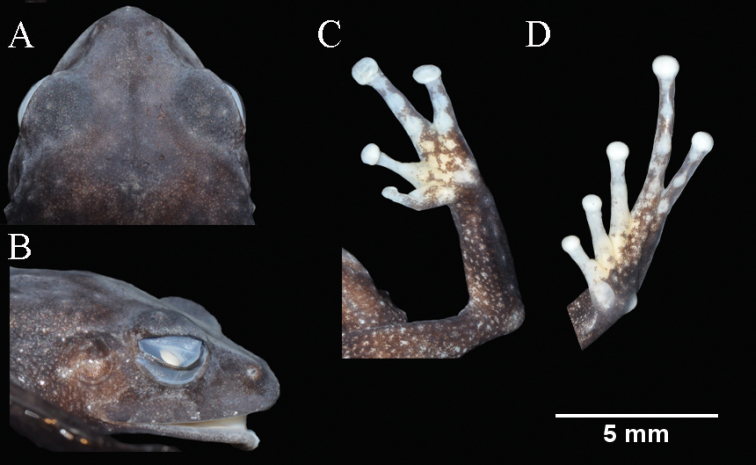
Holotype of *Pristimantisquintanai* sp. nov. in preservative, adult female MZUA.AN.1881. **A** head in dorsal view **B** head in profile view **C** palmar surfaces **D** plantar surfaces.

**Holotype.** MZUA.AN.1881 (Figs 10–12), an adult female collected in Guangras, Rivera perish, Azogues canton, Cañar Province, Ecuador (2.4826S, 78.6019W; datum WGS84), 2527 m above sea level, by Juan C. Sanchez-Nivicela, Amanda Quezada Bruno Timbe and Jhonny Cedeño.

**Paratypes.** Two males MZUA.AN.1880, MZUA.AN.1900, three females MZUA.AN.1873, MZUA.AN.1885, MZUA.AN.1874 and a subadult female MZUA.AN.1890 collected with the holotype. Two females MZUA.AN.1746, MZUA.AN.1748 and a subadult female MZUA.AN.1747 collected from Rivera, Rivera perish, Azogues canton, Cañar Province, Ecuador (2.5459S, 78.6303W; datum WGS84), 2699 m by Juan C. Sanchez-Nivicela, Eduardo Toral and Veronica L. Urgiles and a female MZUA.AN.2705 collected from Llavircay Rivera perish, Azogues canton, Cañar Province, Ecuador (2.5637S, 78.5957W; datum WGS84), 2830 m by Amanda Quezada and Jhonny Cedeño.

########### Diagnosis.

*Pristimantisquintanai* is a small species characterized by: (1) skin of dorsum finely tuberculated with low and rounded tubercles that vary in size (character more noticeable in life), notorious dermal crests, elevated; skin on venter coarsely areolate, dorsolateral folds present, low, middorsal fold low, discoidal fold barely noticeable; low sinusoidal scapular fold; (2) tympanic membrane indistinct, tympanic annulus differentiated, visible, rounded (57% of ED), postrictal tubercles present; (3) short snout, slightly subacuminate in dorsal view, rounded in profile, canthus rostralis concave; (4) upper eyelid with one or two rounded tubercles and with several low ones, cranial crest absent; (5) dentigerous processes of vomer oblique, with one to two teeth, rounded choana; (6) males have small vocal slits but lack vocal sac and nuptial pads; (7) Finger I shorter than Finger II, discs rounded, with dilated pads in all fingers, well defined circumferential grooves; (8) lateral fringes of finger barely noticeable; (9) ulnar tubercles present, lacking antebrachial tubercles; (10) heel with one rounded and several low tubercles, shank lacking tubercles, tarsal tubercles low and small; (11) lateral fringes on toes barely noticeable, webbing absent; Toe V longer than Toe III; discs of toes rounded, dilated pads in all toes, well defined circumferential grooves; (12) inner metatarsal tubercles ovoid two times bigger than outer one, rounded; supernumerary plantar tubercles very low and small, smaller than subarticular tubercles; (13) iris grayish-gold with thin dark reticulations and a horizontal reddish stripe in the middle of the eye, dorsal coloration varies between dark brown, or light brown with cream; flanks vary between dark brown with minute white spots to light cream or yellowish-cream with minute white spots; ventral coloration varies between black, light reddish-brown or reddish-cream; groin and concealed surfaces of thighs are black with white irregular spots (whitish-cream and smaller in males); (14) SVL 19.0–21.8 mm in adult females (20.5 ± 0.90 SD, *N* = 6) and 15.5–16.4 mm in adult males (16.0 ± 0.64 SD, *N* = 2).

########### Comparison with simil﻿ar species.

*Pristimantisquintanai* is morphologically most similar to *P.simonbolivari*, *P.orestes*, and *P.saturninoi* from the *P.orestes* complex. The new species is similar to *P.saturninoi*, *P.orestes* sensu stricto, and *P.cajanuma* and *P.simonbolivari* in having white spots on the groin. However, it can be distinguished from *P.saturninoi* by having expanded discs in fingers and toes (narrower in *P.saturninoi*), by lacking tympanic membrane, and because males lack nuptial pads. The new species differs from *P.orestes* sensu stricto by having a low dorsolateral fold, a ventral coloration that varies between black, reddish-brown or reddish-cream (gray to pale brown spotted with cream and brown in *P.orestes*), and because males lack vocal sacs. *Pristimantisquintanai* is different from *P.cajanuma* by having a finely tuberculated dorsal skin and a ventral coloration that can vary between black, reddish-brown or reddish-cream (light gray with or without dark flecks and skin texture shagreen in *P.cajanuma*). *Pristimantisquintanai* differs from *P.simonbolivari* by having a finely tuberculated dorsal skin (smooth in *P.simonbolivari*), males with vocal sacs, and a row of ulnar tubercles (indistinct in *P.simonbolivari*).

*Pristimantisbambu* is different from the new species by having a finely granular dorsal skin, ulnar tubercles coerced into a fold, vocal sacs in males (absent in *P.quintanai*), yellow coloration in the groin, and by lacking tubercles on the heel (one small rounded and several low in *P.quintanai*). *Pristimantismazar* is different by lacking tubercles on the upper eyelid (one or two small rounded and several low in *P.quintanai*) and by having a well differentiated tympanic membrane, a dark reticulated pattern in the groin, a creamish-gray to dark brownish gray dorsal coloration and a whitish-cream coloration in the venter. *Pristimantisandinognomus* is different from the new species by having enlarged conical tubercles on heel and upper eyelids (one or two rounded and several low in *P.quintanai*), a differentiated tympanic membrane, males with vocal sacs and pale cupper dorsal coloration. *Pristimantisvidua* is different by having a finely granular dorsal skin and by lacking ulnar tubercles. Finally, *P.tiktik* is different by lacking dorsolateral folds (present, low in *P.quintanai*) and because males have vocal sacs, a reddish coloration on the groin (irregular white or whitish-cream in *P.quintanai*) and a ventral coloration that varies between various shades of gray, brown, orange or green (black, reddish-brown or reddish-cream in *P.quintanai*).

########### Description of holotype.

Adult female (Figs 10–12) with head narrower than body and wider than long. HL is 87% of HW, HW 36% of SVL; HL 31% of SVL; snout short (snout to eye distance 6% of SVL), subacuminate in dorsal view, rounded in profile (Fig. 12A, B); canthus rostralis concave in dorsal view, angular in profile; loreal region flat; ED 60% of eye-nostril distance; nostrils oriented laterally; lips not flared; cranial crests absent; upper eyelid bearing one small subconical tubercle and low small tubercles, width of upper eyelid 57% of IOD; tympanic annulus, rounded, its upper and posterodorsal part obscured by a low and short supratympanic fold; tympanic membrane absent (Fig. 12B); diameter of tympanum 63% of ED; one postrictal tubercle posteroventral to the tympanic annulus; choanae small, round, no concealed by palatal shelf of maxillary arch; dentigerous processes of vomers triangular, slightly larger than the choanae, without space between the processes, bearing one teeth on the left one and two teeth on the right one; tongue 1.4× as long as wide, slightly notched posteriorly, posterior half not adherent to floor of mouth.

Skin on dorsum finely tuberculated; middorsal fold present; low dorsolateral folds (more noticeable toward the end of dorsum); sinusoidal scapular fold present (Fig. 11A) skin of throat shagreen with few small scattered tubercles, skin on chest and belly coarsely areolate; discoidal fold low, barely noticeable (Fig. 11B); cloacal region with enlarged warts.

Ulnar tubercles present, outer palmar tubercle bifurcated (divided distally); thenar tubercle rounded; subarticular tubercles not projected, round and subconical in section; supernumerary palmar tubercles low and rounded, smaller than subarticular tubercles; fingers bearing lateral fringes; Finger I shorter than Finger II; discs on fingers laterally expanded, rounded; all fingers bearing dilated pads well defined by circumferential grooves (Fig. 12C).

Hindlimbs short; TL and FL are 40% of SVL; heel with two small subconical tubercles (the one closest to the tarsus bigger); outer edge of tarsus with a row of small and low tubercles; inner edge of tarsus bearing a fold; inner metatarsal tubercle broadly ovoid, about 2× the rounded outer metatarsal tubercle; subarticular tubercles not projected; plantar supernumerary tubercles low, barely noticeable; toes bearing lateral fringes; webbing absent; discs on toes laterally expanded, rounded, wider than those on fingers; toes with dilated pads well defined by circumferential grooves; relative length of toes I <II < III < V < IV (Fig. 12D).

########### Measurements of holotype.

SVL 20.2; HW 7.2; HL 6.3; IOD 2.6; internarial distance 1.8; upper EW 1.5; ED 2.2; eye-nostril distance 1.3; TD 1.4; TL 8.8; FL 8.5.

########### Coloration of holotype.

In life (Fig. 10) the dorsum is brown, but it becomes darker toward the flanks. The tips of the tubercles, that cover most of the dorsal surfaces, are slightly pinkish. A dark brown strip is visible in the supratympanic region. The loreal region, nostrils and upper lips have vertical dark brown chevrons. The dorsal surfaces of finger tips are dark cream. The throat is dark brown with minute pinkish-cream spots, the venter is dark brown, the groin and concealed surfaces of the thighs and tibia are black with irregular white spots (larger in the groin region). The venter is black. The ventral surfaces of hands are cream with dark brown spots. Toes I, II and III and the tips of Toes IV and V are cream, the plantar surfaces as well as Toes IV and V present a dense brown spatter. The iris is grayish-gold with dark reticulations and a reddish horizontal bar in the middle. The cloacal region presents a dark triangle delimited by a thin gray strip that extends to the thighs.

In preservative (Fig. 11) the dorsum and flanks are dark brown with tiny light brown dots (the tip of the tubercles is light gray). The head and upper eyelids are grayish-brown, the dorsal surfaces of the limbs present the same coloration as the dorsum. The dorsal surfaces of hands and foot are light brown with cream spots, the dorsal surfaces of the tips in Fingers I and II are cream. The dorsal surfaces of toes I, II and III are cream with a tiny brown spatter. The throat and chest are light brown, the venter is dark brown, the groin and concealed surfaces of thighs and tibia are dark brown with white irregular spots. The ventral surfaces of hands are white whit brown spatter. Toes I, II and III and the tips of Toes IV and V are white, the plantar surface as well as Toes IV and V show a dense brown spatter. The cloacal region presents a dark triangle delimited by a thin gray strip that extends to the thighs.

########### Variation.

**Figure 13.**
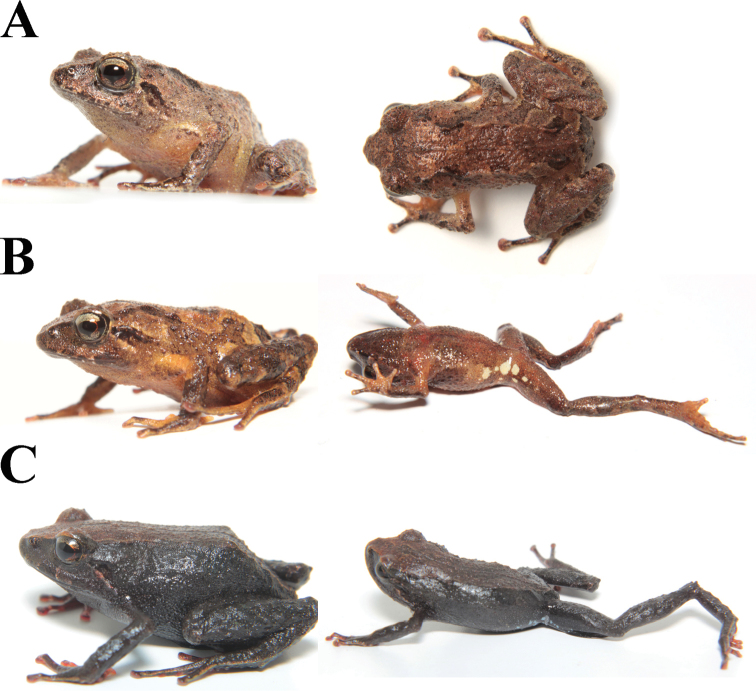
Morphological variation of *Pristimantisquintanai* sp. nov. in live. **A** MZUA.AN.1900, profile and dorsal view **B** MZUA.AN.1880, profile and ventral view **C** MZUA.AN.2705, profile view.

Morphometric variation is detailed in Table 5. In the males MZUA.AN.1900 (Fig. 13A) and MZUA.AN.1880 (Fig. 13B), the tubercles on the dorsum and on the upper eyelid are less distinguishable (character more notorious in life in these specimens). One individual, MZUA.AN.2705 (Fig. 13C) has smaller blueish-white spots on the groin, the dorsal surfaces of finger tips and toes and the ventral surfaces of hands and foot are pink. The throat is dark brown with minute dark gray spots. In the male MZUA.AN.1900, the flanks and posterior limbs have dark brown vertical chevrons delimited by cream. The throat, chest and the region of the flanks next to the belly is yellowish-cream, the venter is reddish-cream. The male, MZUA.AN.1880 presents a lighter dorsal coloration with a light brown and yellowish-cream pattern.

**Table 5.** Measurements (in mm) of adult males and females of *Pristimantisquintanai* sp. nov. The mean, standard deviation (SD) and range of each morphological character are shown for females (*N* = 6). The mean of each character is show for males (*N* = 2). Abbreviations of the morphometric measurements are presented in Materials and methods.

	**MZUA**								**Mean** ± **SD (range)** ♀	**Mean** ♂
	**1881** ♀	**1746** ♀	**1885** ♀	**1873** ♀	**2705** ♀	**1874** ♀	**1900** ♂	**1880** ♂		
**SVL**	20.2	20.7	20.6	21.8	19.0	20.6	15.5	16.4	20.5 ± 0.9 (19.0–21.8)	16.0
**EN**	1.3	1.2	1.7	1.9	1.7	1.6	1.0	1.1	1.6 ± 0.3 (1.2–1.9)	1.1
**TD**	1.4	1.4	1.1	1.1	1.1	1.1	0.8	0.9	1.2 ± 0.2 (1.1–1.4)	0.9
**ED**	2.2	2.2	2.1	2.1	2.0	2.1	1.7	1.7	2.1 ± 0.1 (2.0–2.2)	1.7
**EW**	1.5	1.7	1.4	1.8	1.6	1.6	1.0	1.3	1.6 ± 0.1 (1.5–1.8)	1.2
**IOD**	2.6	2.4	2.8	3.0	2.5	2.5	1.9	2.0	2.6 ± 0.2 (2.4–3.0)	2.0
**IND**	1.8	2.0	2.0	2.0	1.7	1.8	1.4	1.7	1.9 ± 0.1 (1.8-–.0)	1.6
**HL**	6.3	6.5	6.5	7.0	5.9	6.2	4.5	4.8	6.4 ± 0.4 (5.9–6.5)	4.7
**HW**	7.2	7.4	7.5	7.8	6.8	7.2	5.6	5.8	7.3 ± 0.3 (6.8–7.0)	5.7
**TL**	8.8	9.0	9.4	9.5	8.8	8.9	7.0	7.4	9.1 ± 0.3 (8.8–9.5)	7.2
**FL**	8.5	7.7	8.5	8.9	8.4	8.7	6.5	7.2	8.5 ± 0.4 (7.7–8.9)	6.9

########### Etymology.

The specific epithet honors Dr Pedro Quintana-Ascencio for his contributions teaching young scientists from Ecuador and the USA and for promoting conservation studies in endangered ecosystems in the south of Ecuador. This is our tribute to Pedro as an ecologist, professor and friend.

########### Distribution and natural history.

*Pristimantisquintanai* is know from three localities in the Province of Cañar: Guangras, Rivera and Llavircay in an elevation range between 2500 and 2800 m. The ecosystem were the species is found is categorized as an evergreen high montane forest from the eastern Andes of Ecuador (Ministerio de Ambiente del Ecuador 2012). All specimens were encountered during the night between small shrubs and in leaf litter. Some specimens were observed in small branches between 0 and 25 cm above ground. Other sympatric frogs include *P.pycnodermis* and two other unidentified species of *Pristimantis*.

########### Conservation status.

The localities where *P.quintanai* has been registered cover an estimated area of 40 km^2^. The landscape is highly fragmented and includes extensive areas of both active and abandoned paddocks and has been directly influenced by the infrastructure of the Mazar hydroelectric project. In all the localities, the montane forest has been drastically reduced, particularly next to villages and cities. *Pristimantisquintanai* is not a locally abundant species given that only a handful of individuals (<7) were found in each of the visited localities. We therefore recommend that this species be categorized as Endangered B1ab (iii), following the IUCN criteria, because its extent of occurrence is less than 5000 km^2^ and its natural habitat has been severely fragmented.

## Discussion

Recent phylogenies published by Brito et al. (2017) and Székely et al. (2018) have advanced our understanding of the *P.orestes* species group but continue to recover *P.orestes* as three different lineages from three localities of southern Ecuador: 1) Cajanuma (Loja), 2) Lagunas del Compadre (Loja) and 3) Sigsig (Azuay). The issue of paraphyly arises from a lack of molecular data from the type species that was collected in 1971 by William E. Duellman and Bruce MacBride (Lynch 1979) in the locality of Urdaneta, province of Loja. In our study, we provide for the first time genetic sequences of *P.orestes* sensu stricto from four specimens collected at the type locality. Notably, the *P.orestes* specimens from Urdaneta cluster together with the specimen from Sigsig in a strongly supported clade. Thus, in our analysis, we found no evidence to suggest that the individual from the Sigsig locality is genetically distinct from those in Urdaneta and therefore we maintain the identity of KU18257 as *P.orestes*. In contrast, we found evidence to suggest that the specimen from the nearby locality Lagunas del Compadre is genetically distinct and should therefore not be considered part of *P.orestes* sensu stricto. We aim to provide a complete description of the new species from Lagunas del Compadre based on a larger number of specimens in a future study.

Our analyses show that *Pristimantissaturninoi* consists of two genetically distinct lineages. One lineage includes the holotype and one paratype from the description of Brito et al. (2017), and as such, we consider this clade as *P.saturninoi* sensu stricto. A second paratype (DHMECN 12237) clusters together with *P.quintanai* in a distinct clade and therefore should be considered as a distinct species from *P.saturninoi.* Although we find a moderate genetic distance (2.3%, based on the 16S fragment) between DHMECN 12237 and *P.quintanai*, we still need genetic, morphological and behavioral evidence (i.e., calls) from a larger number of individuals to determine relationships with *P.quintanai*. This conflict within *P.saturninoi* is most likely the result of convergent morphological resemblance between the collected specimens that prevents their separation based on morphological characteristics only. Similar issues with type series that are found to consist of different species have been reported in other clades within *Pristimantis* (Ortega-Andrade and Venegas 2014), highlighting the importance of obtaining different lines of evidence including genetic, morphological and ecological data when dealing with complex cryptic groups of species (Araujo De Oliveira et al. 2017) such as the *P.orestes* species group.

A handful of morphological characters including the characteristic white spots in the groin are shared between *P.orestes* and the newly described species *P.cajanuma* and *P.quintanai*. Here, we find evidence of strong genetic differentiation between these species and provide a combination of additional morphological characters that can help to easily distinguish between these species in the field. Our phylogeny suggests that true diversity within the *P.orestes* species group is yet to be fully uncovered, and that formal descriptions for several new taxa (e.g., DHMECN 3112, QCAZ 45556) are still needed. Moreover, as detailed here for *P.saturninoi* and *P.orestes* sensu stricto, additional genetic data are also needed from other potential members such as *P.colodactylus*, *P.vidua*, and *P.tinajillas* to infer the evolutionary history of the group. As we conduct more field expeditions in the southern highlands of the Ecuadorean Andes with a focus on the type localities, we are confident that the diversity as well as our understanding of phylogenetic relationships of the *P.orestes* species group will significantly increase.

## Supplementary Material

XML Treatment for Pristimantis orestes

XML Treatment for Pristimantis cajanuma

XML Treatment for Pristimantis quintanai

## Supplementary materials

Supplementary material 1 Specimens used for morphological comparisons Data type: species data Explanation note: For each specimen we present the museum number and locality. https://binary.pensoft.net/file/318822This dataset is made available under the Open Database License (http://opendatacommons.org/licenses/odbl/1.0/). The Open Database License (ODbL) is a license agreement intended to allow users to freely share, modify, and use this Dataset while maintaining this same freedom for others, provided that the original source and author(s) are credited.Veronica L. Urgiles, Paul Székely, Diana Székely, Nicholas Christodoulides, Juan C. Sanchez-Nivicela, Anna E. Savage

Supplementary material 2 Advertisement call of *Pristimantis orestes* sensu stricto Data type: species data https://binary.pensoft.net/file/318823This dataset is made available under the Open Database License (http://opendatacommons.org/licenses/odbl/1.0/). The Open Database License (ODbL) is a license agreement intended to allow users to freely share, modify, and use this Dataset while maintaining this same freedom for others, provided that the original source and author(s) are credited.Veronica L. Urgiles, Paul Székely, Diana Székely, Nicholas Christodoulides, Juan C. Sanchez-Nivicela, Anna E. Savage

Supplementary material 3 Data of call recordings used in the present study Data type: species data Explanation note: Dist. = distance from the focal male; Call = call duration; Temp. = air temperature; H. = air humidity. https://binary.pensoft.net/file/318824This dataset is made available under the Open Database License (http://opendatacommons.org/licenses/odbl/1.0/). The Open Database License (ODbL) is a license agreement intended to allow users to freely share, modify, and use this Dataset while maintaining this same freedom for others, provided that the original source and author(s) are credited.Veronica L. Urgiles, Paul Székely, Diana Székely, Nicholas Christodoulides, Juan C. Sanchez-Nivicela, Anna E. Savage

Supplementary material 4 Single gene trees for 12S, 16S and RAG-1 for the *Pristimantis orestes* species group, inferred with Maximum Likelihood Data type: molecular data Explanation note: Bootstrap support is shown for nodes over 70%. https://binary.pensoft.net/file/318825This dataset is made available under the Open Database License (http://opendatacommons.org/licenses/odbl/1.0/). The Open Database License (ODbL) is a license agreement intended to allow users to freely share, modify, and use this Dataset while maintaining this same freedom for others, provided that the original source and author(s) are credited.Veronica L. Urgiles, Paul Székely, Diana Székely, Nicholas Christodoulides, Juan C. Sanchez-Nivicela, Anna E. Savage

Supplementary material 5 Maximum Likelihood tree inference of the *Pristimantis orestes* species group Data type: statistical data Explanation note: Bootstrap support values are shown for nodes over 70%. The bar coloration indicates the following: *P. cajanuma* sp. nov. (yellow), *P. orestes* (dark red), *P. saturninoi* (blue), and *P. quintanai* sp. nov. (green). https://binary.pensoft.net/file/318826This dataset is made available under the Open Database License (http://opendatacommons.org/licenses/odbl/1.0/). The Open Database License (ODbL) is a license agreement intended to allow users to freely share, modify, and use this Dataset while maintaining this same freedom for others, provided that the original source and author(s) are credited.Veronica L. Urgiles, Paul Székely, Diana Székely, Nicholas Christodoulides, Juan C. Sanchez-Nivicela, Anna E. Savage

## References

[B1] Arteaga AF Guayasamin JM (2011) A new frog of the genus *Pristimantis* (Amphibia: Strabomantidae) from the high Andes of southeastern Ecuador, discovered using morphological and molecular data. Zootaxa 2876: 17–29. 10.11646/zootaxa.3616.4.3

[B2] Boulenger GA (1900) Descriptions of new batrachians and reptiles collected by Mr. P. O. Simons in Peru. Annals and Magazine of Natural History, Series 7(6): 181–186. 10.1080/00222930008678355

[B3] Brito J Almendariz A Batallas D Ron SR (2017) Nueva especie de rana bromelícola del género *Pristimantis* (Amphibia: Craugastoridae), meseta de la cordillera del Cóndor, Ecuador. Papeis Avulsos de Zoologia 57(15): 177–195. 10.11606/0031-1049.2017.57.15

[B4] Brito J Batallas D Yanez-Munoz MH (2017) Ranas terrestres *Pristimantis* (Anura: Craugastoridae) de los bosques montanos del rio Upano, Ecuador: Lista anotada, patrones de diversidad y descripción de cuatro especies nuevas. Neotropical Biodiversity 3: 125–156. 10.1080/23766808.2017.1299529

[B5] Cocroft RB Ryan MJ (1995) Patterns of advertisement call evolution in toads and chorus frogs. Animal Behaviour 49: 283–303. 10.1006/anbe.1995.0043

[B6] Darst CR Cannatella DC (2004) Novel relationships among hyloid frogs inferred from 12S and 16S mitochondrial DNA sequences. Molecular Phylogenetics and Evolution 31: 462–475. 10.1016/j.ympev.2003.09.003 15062788

[B7] Duellman WE Lehr E (2009) Terrestrial-breeding frogs (Strabomantidae) in Peru. Nature und Tier Verlag, Münster, 382 pp.

[B8] Duellman WE Pramuk JB (1999) Frogs of the genus *Eleutherodactylus* (Anura: Leptodactylidae) in the Andes of northern Peru. Scientific Papers. Natural History Museum, University of Kansas 13: 1–78. 10.5962/bhl.title.16169

[B9] Guayasamin JM Arteaga AF (2013) A new species of the Pristimantisorestes group Amphibia: Strabomantidae) from the high Andes of Ecuador, Reserva Mazar, Zootaxa. 3616(4): 345–356. 10.11646/zootaxa.3616.4.3 24758815

[B10] Guayasamin JM Arteaga A Hutter CR (2018) A new (singleton) rainfrog of the *Pristimantismyersi* Group (Amphibia: Craugastoridae) from the northern Andes of Ecuador. Zootaxa, 4527(3): 323–334. 10.11646/zootaxa.4527.3.2 30651427

[B11] Guayasamin JM Hutter CR Tapia EE Culebras J Peñafiel N Pyron RA Morochz C Funk C Arteaga A (2017) Diversification of the rainfrog *Pristimantisornatissimus* in the lowlands and Andean foothills of Ecuador. PLoS ONE 12(3): e0172615. 10.1371/journal.pone.0172615 PMC5362048 28329011

[B12] Hedges SB Duellman WE Heinicke MP (2008) New World direct-developing frogs (Anura: Terrarana): molecular phylogeny, classification, biogeography, and conservation. Zootaxa 1737: 1–182. 10.11646/zootaxa.3986.2.1

[B13] Heinicke MP Duellman WE Hedges SB (2007) Major Caribbean and Central American frog faunas originated by ancient oceanic dispersal. Proceedings of the National Academy of Sciences 104(24): 10092–10097. 10.1073/pnas.0611051104 PMC1891260 17548823

[B14] IUCN (2018) The IUCN Red List of Threatened Species. Version 2018-2. http://www.iucnredlist.org

[B15] Jiménez de la Espada M (1870) Fauna neotropicalis species quaedam nondum cognitae. Jornal de Sciências, Mathemáticas, Physicas e Naturaes 3: 57–65.

[B16] Katoh K Standley DM (2013) MAFFT multiple sequence alignment software version 7: improvements in performance and usability. Molecular Biology and Evolution 4: 772–80. 10.1093/molbev/mst010 PMC3603318 23329690

[B17] Kieswetter CM Schneider CJ (2013) Phylogeography in the northern Andes: complex history and cryptic diversity in a cloud forest frog, *Pristimantisw-nigrum* (Craugastoridae). Molecular Phylogenetics and Evolution. 69(3): 417–29. 10.1016/j.ympev.2013.08.007 23978627

[B18] Köhler J Jansen M Rodríguez A Kok PJR Toledo LF Emmrich M Glaw F Haddad CFB Rödel MO Vences M (2017) The use of bioacoustics in anuran taxonomy: theory, terminology, methods and recommendations for best practice. Zootaxa 4251: 1–124. 10.11646/zootaxa.4251.1.1 28609991

[B19] Kumar S Stecher G Li M Knyaz C Tamura K (2018) MEGA X: Molecular Evolutionary Genetics Analysis across Computing Platforms, Molecular Biology and Evolution 35(6): 1547–1549. 10.1093/molbev/msy096 PMC5967553 29722887

[B20] Lanfear R Frandsen PB Wright AM Senfeld T Calcott B (2016) PartitionFinder 2: new methods for selecting partitioned models of evolution for molecular and morphological phylogenetic analyses. Molecular biology and evolution. 10.1093/molbev/msw260 28013191

[B21] Lehr E Coloma LA (2008) A minute new Ecuadorian Andean frog (Anura: Strabomantidae, *Pristimantis*). Journal of Herpetology 64(3): 354–367. 10.1655/07-089.1

[B22] Lynch JD (1979) Leptodactylid frogs of the genus *Eleutherodactylus* from the Andes of southern Ecuador. Miscellaneous Publications, Museum of Natural History, University of Kansas 66: 1–62. 10.5962/bhl.title.16268

[B23] Lynch JD Duellman WE (1997) Frogs of the genus *Eleutherodactylus* in western Ecuador: systematics, ecology, and biogeography. Special Publication Natural History Museum University of Kansas 23: 1–236. 10.5962/bhl.title.7951

[B24] McDiarmid RW (1994) Preparing amphibians as scientific specimens. In: Heyer WR, Donnelly MA, McDiarmid RW, Hayek LC, Foster MS (Eds) Measuring and Monitoring Biological Diversity Standard Methods for Amphibians, Smithsonian Press, Washington, DC, 289–297.

[B25] Miller MA Pfeiffer W Schwartz T (2010) “Creating the CIPRES Science Gateway for inference of large phylogenetic trees” in Proceedings of the Gateway Computing Environments Workshop (GCE), New Orleans, LA, 14 November 2010, 1–8. 10.1109/GCE.2010.5676129

[B26] Ministerio de Ambiente del Ecuador (2012) Sistema de Clasificación de los Ecosistemas del Ecuador Continental. Subsecretaría de Patrimonio Natural, Quito, 143 pp.

[B27] Nguyen LT Schmidt HA von Haeseler A Minh BQ (2015) IQ-TREE: a fast and effective stochastic algorithm for estimating maximum-likelihood phylogenies. Molecular Biology and Evolution 32(1): 268–74. 10.1093/molbev/msu300 25371430 PMC4271533

[B28] Oliveira EA Rodrigues LR Kaefer IL Pinto KC Hernández-Ruz EJ (2017) A new species of *Pristimantis* from eastern Brazilian Amazonia (Anura, Craugastoridae). Zookeys 687: 101–29. 10.3897/zookeys.687.13221 PMC5672576 29114168

[B29] Ortega-Andrade HM Venegas PJ (2014) A new synonym for *Pristimantisluscombei* (Duellman and Mendelson 1995) and the description of a new species of *Pristimantis* from the upper Amazon basin (Amphibia: Craugastoridae). Zootaxa 3895(1): 31–57. 10.11646/zootaxa.3895.1.2 25543553

[B30] Padial JM Grant T Frost DR (2014) Molecular systematics of terraranas (Anura: Brachycephaloidea) with an assessment of the effects of alignment and optimality criteria. Zootaxa 3825(1): 1–132. 10.11646/zootaxa.3825.1.1 24989881

[B31] Palumbi SR Martin A Romano S McMillan WO Stice L Grabowski G (1991) The Simple Fool’s Guide to PCR. Version 2.0. University of Hawaii, Honolulu.

[B32] Pinto-Sanchez NR Ibanez R Madrinan S Sanjur OI Bermingham E Crawford AJ (2012) The Great American Biotic Interchange in frogs: multiple and early colonization of Central America by the South American genus *Pristimantis* (Anura: Craugastoridae). Molecular Phylogenetics and Evolution 62: 954–972. 10.1016/j.ympev.2011.11.022 22178362

[B33] Pyron RA Wiens JJ (2011) A large-scale phylogeny of Amphibia with over 2,800 species, and a revised classification of extant frogs, salamanders, and caecilians. Molecular Phylogenetics and Evolution 61: 543–583. 10.1016/j.ympev.2011.06.012 21723399

[B34] Rambaut A Suchard MA Xie D Drummond AJ (2014) Tracer v1.5, Available from: http://beast.bio.ed.ac.uk/Tracer

[B35] Ron SR Merino-Viteri A Ortiz DA (2019) Anfibios del Ecuador. Version 2019.0. Museo de Zoología, Pontificia Universidad Católica del Ecuador. https://bioweb.bio/faunaweb/amphibiaweb

[B36] Ronquist F Teslenko M van der Mark P Ayres DL Darling A Hohna S Larget B Liu L Suchard MA Huelsenbeck JP (2012) MrBayes 3.2: efficient Bayesian phylogenetic inference and model choice across a large model space. Systematics Biology 61(3): 539–42. 10.1093/sysbio/sys029 PMC3329765 22357727

[B37] Székely P Eguiguren JS Székely D Ordonez-Delgado L Armijos-Ojeda D Riofrio-Guaman ML Cogălniceanu D (2018) A new minute *Pristimantis* (Amphibia: Anura: Strabomantidae) from the Andes of southern Ecuador. PLoS ONE 13(8): e0202332. 10.1371/journal.pone.0202332 PMC6114709 30157209

[B38] Toledo LF Martins IA Bruschi DP Passos MA Alexandre C Haddad CFB (2015) The anuran calling repertoire in the light of social context. Acta Ethologica 18: 87–99. 10.1007/s10211-014-0194-4

[B39] Watters JL Cummings ST Flanagan RL Siler CD (2016) Review of morphometric measurements used in anuran species descriptions and recommendations for a standardized approach. Zootaxa 4072(4): 477–495. 10.11646/zootaxa.4072.4.6 27395941

[B40] Wiens JJ Coloma LA (1992) A new species of the *Eleutherodactylusmyersi* (Anura: Leptodactylidae) assembly from Ecuador. Journal of Herpetology 26: 196–207. 10.2307/1564862

