# Supplementary material for: Genetic delimitation of Pristimantisorestes (Lynch, 1979) and P.saturninoi Brito et al., 2017 and description of two new terrestrial frogs from the Pristimantisorestes species group (Anura, Strabomantidae)
Source: Zookeys. 2019 Jul 18;864:111–46. doi: 10.3897/zookeys.864.35102 (PMC6658573; doi:10.3897/zookeys.864.35102)
Supplement: Supplementary material 1 [file zookeys-864-111-s001.docx]

**Supplementary material 1.** Specimens used for morphological comparisons. For each specimen we present the museum number and locality.

| **Museum number** | **Species** | **Locality** |
| --- | --- | --- |
| DHMECN9667 | *Pristimantis andinognomus* | Ecuador: Zamora Chinchipe, Urdaneta |
| DHMECN9677 | *Pristimantis andinognomus* | Ecuador: Zamora Chinchipe, Urdaneta |
| DHMECN9683 | *Pristimantis andinognomus* | Ecuador: Zamora Chinchipe, Urdaneta |
| QCAZ46740 | *Pristimanti bambu* | Ecuador: Cañar, La Libertad, Reserva Mazar |
| QCAZ46704 | *Pristimanti bambu* | Ecuador: Cañar, La Libertad, Reserva Mazar |
| QCAZ46710 | *Pristimanti bambu* | Ecuador: Cañar, La Libertad, Reserva Mazar |
| KU142151 | *Pristimantis colodactylus* | Ecuador: Loja, Abra del Zamora |
| KU142160 | *Pristimantis colodactylus* | Ecuador: Loja, Abra del Zamora |
| QCAZ68408 | *Pristimantis mazar* | Ecuador: Cañar, La libertad |
| QCAZ68403 | *Pristimantis mazar* | Ecuador: Cañar, La libertad |
| QCAZ68390 | *Pristimantis mazar* | Ecuador: Cañar, La libertad |
| QCAZ68393 | *Pristimantis mazar* | Ecuador: Cañar, La libertad |
| QCAZ68368 | *Pristimantis mazar* | Ecuador: Cañar, La libertad |
| QCAZ68406 | *Pristimantis mazar* | Ecuador: Cañar, La libertad |
| QCAZ68384 | *Pristimantis mazar* | Ecuador: Cañar, La libertad |
| QCAZ27556 | *Pristimantis mazar* | Ecuador: Cañar, La libertad |
| QCAZ27553 | *Pristimantis mazar* | Ecuador: Cañar, La libertad |
| QCAZ27554 | *Pristimantis mazar* | Ecuador: Cañar, La libertad |
| QCAZ27493 | *Pristimantis mazar* | Ecuador: Cañar, Rumiloma, Reserva Mazar |
| QCAZ 65025 | *Pristimantis muranunka* | Ecuador: Zamora Chinchipe, Nuevo Paraiso |
| QCAZA40783 | *Pristimantis orestes* | Ecuador: Azuay, Sigsig |
| KU141998 | *Pristimantis* sp. | Ecuador: Loja, Urdaneta |
| KU142002 | *Pristimantis* sp. | Ecuador: Loja, Urdaneta |
| KU14200 | *Pristimantis* sp. | Ecuador: Loja, Urdaneta |
| QCAZ 45556 | *Pristimantis* sp. | Ecuador: Loja, Lagunas del Compadre |
| DHMECN 12232 | *Pristimantis saturninoi* | Ecuador: Morona Santiago, Cerro Sambalan, |
| DHMECN 12235 | *Pristimantis saturninoi* | Ecuador: Morona Santiago, Cerro Sambalan, |
| DHMECN 12231 | *Pristimantis saturninoi* | Ecuador: Morona Santiago, Cerro Sambalan, |
| DHMECN12233 | *Pristimantis saturninoi* | Ecuador: Morona Santiago, Cerro Sambalan, |
| DHMECN12234 | *Pristimantis saturninoi* | Ecuador: Morona Santiago, Cerro Sambalan, |
| DHMECN12237 | *Pristimantis saturninoi* | Ecuador: Morona Santiago, Cerro Sambalan, |
| DHMECN12238 | *Pristimantis saturninoi* | Ecuador: Morona Santiago, Cerro Sambalan, |
| DHMECN12247 | *Pristimantis saturninoi* | Ecuador: Morona Santiago, Cerro Sambalan, |
| DHMECN 10745 | *Pristimantis tinajillas* | Ecuador: Morona Santiago, Tinajillas |
| DHMECN 10746 | *Pristimantis tinajillas* | Ecuador: Morona Santiago, Tinajillas |
| MZUA.An0968 | *Pristimantis tinajillas* | Ecuador: Morona Santiago, Tinajillas |
| MZUA.An0969 | *Pristimantis tinajillas* | Ecuador: Morona Santiago, Tinajillas |
| MZUA.An1126 | *Pristimantis tinajillas* | Ecuador: Morona Santiago, Tinajillas |
| MZUA.An1127 | *Pristimantis tinajillas* | Ecuador: Morona Santiago, Tinajillas |
| QCAZ37665 | *Pristimantis simonbolivari* | Ecuador: Bolivar, Bosque Protector Cashca Totoras |
| QCAZ37666 | *Pristimantis simonbolivari* | Ecuador: Bolivar, Bosque Protector Cashca Totoras |
| QCAZ37667 | *Pristimantis simonbolivari* | Ecuador: Bolivar, Bosque Protector Cashca Totoras |
| QCAZA57374 | *Pristimantis simonbolivari* | Ecuador: Bolivar, Bosque Protector Cashca Totoras |
| QCAZA57375 | *Pristimantis simonbolivari* | Ecuador: Bolivar, Bosque Protector Cashca Totoras |
| QCAZA64884 | *Pristimantis simonbolivari* | Ecuador: Bolivar, Bosque Protector Cashca Totoras |
| MZUA.AN.2492 | *Pristimantis tiktik* | Ecuador: Loja, Urdaneta |
| MZUA.AN.2495 | *Pristimantis tiktik* | Ecuador: Loja, Urdaneta |
| MZUA.AN.2496 | *Pristimantis tiktik* | Ecuador: Loja, Urdaneta |
| MZUA.AN.2498 | *Pristimantis tiktik* | Ecuador: Loja, Urdaneta |
| MUTPL239 | *Pristimantis tiktik* | Ecuador: Loja, Urdaneta |
| MUTPL251 | *Pristimantis tiktik* | Ecuador: Loja, Urdaneta |
| MUTPL245 | *Pristimantis tiktik* | Ecuador: Loja, Urdaneta |
| KU120082 | *Pristimantis vidua* | Ecuador: Zamora Chinchipe, 15 Km E Loja |
| KU120090 | *Pristimantis vidua* | Ecuador: Zamora Chinchipe, 15 Km E Loja |
| MUTPL147 | *Pristimantis vidua* | Ecuador: Loja, Bosque Protector Washapamba |
| MUTPL148 | *Pristimantis vidua* | Ecuador: Loja, Bosque Protector Washapamba |
| MUTPL156 | *Pristimantis vidua* | Ecuador: Loja, Bosque Protector Washapamba |
| MUTPL173 | *Pristimantis vidua* | Ecuador: Loja, Bosque Protector Washapamba |
| MUTPL143 | *Pristimantis vidua* | Ecuador: Loja, Bosque Protector Washapamba |
| MUTPL146 | *Pristimantis vidua* | Ecuador: Loja, Bosque Protector Washapamba |
| MUTPL152 | *Pristimantis vidua* | Ecuador: Loja, Bosque Protector Washapamba |
