# Supplementary material for: Genetic delimitation of Pristimantisorestes (Lynch, 1979) and P.saturninoi Brito et al., 2017 and description of two new terrestrial frogs from the Pristimantisorestes species group (Anura, Strabomantidae)
Source: Zookeys. 2019 Jul 18;864:111–46. doi: 10.3897/zookeys.864.35102 (PMC6658573; doi:10.3897/zookeys.864.35102)
Supplement: Supplementary material 3 [file zookeys-864-111-s003.docx]

**Supplementary material 3**. Data of call recordings used in the present study (Dist. = distance from the focal male; Call = call duration; Temp. = air temperature; H. = air humidity).

| **Fonoteca UTPL ID** | **Voucher** | **Locality** | **Coordinates** | **Altitude (m)** | **Date** | **Time** | **Call (min)** | **Dist. (m)** | **Temp. (°C)** | **H. (%)** |
| --- | --- | --- | --- | --- | --- | --- | --- | --- | --- | --- |
|  |  |  |  |  |  |  |  |  |  |  |
| FUTPL-A-129 | no | Ecuador, Loja province, Saraguro canton, 11 km NE of Urdaneta | 3.59154° S 79.13584° W | 2961 | 2016.08.04 | 20:51 | 1:03 | 1 | 10 | 82 |
| FUTPL-A-130 | no | Ecuador, Loja province, Saraguro canton, 11 km NE of Urdaneta | 3.59154° S 79.13584° W | 2961 | 2016.08.04 | 20:55 | 2:02 | 1 | 10 | 83 |
| FUTPL-A-131 | no | Ecuador, Loja province, Saraguro canton, 11 km NE of Urdaneta | 3.59154° S 79.13584° W | 2961 | 2016.08.04 | 20:57 | 0:34 | 1 | 10 | 83.5 |
| FUTPL-A-132 | MUTPL 248 | Ecuador, Loja province, Saraguro canton, 11 km NE of Urdaneta | 3.59153° S 79.13578° W | 2959 | 2016.08.04 | 21:04 | 1:05 | 1 | 9.5 | 83 |
| FUTPL-A-133 | MUTPL 249 | Ecuador, Loja province, Saraguro canton, 11 km NE of Urdaneta | 3.59153° S 79.13578° W | 2959 | 2016.08.04 | 21:16 | 1:13 | 0.5 | 9.5 | 83.5 |
