# Supplementary material for: Genetic delimitation of Pristimantisorestes (Lynch, 1979) and P.saturninoi Brito et al., 2017 and description of two new terrestrial frogs from the Pristimantisorestes species group (Anura, Strabomantidae)
Source: Zookeys. 2019 Jul 18;864:111–46. doi: 10.3897/zookeys.864.35102 (PMC6658573; doi:10.3897/zookeys.864.35102)

### Supplementary material 4. Single gene trees for 12S, 16S and RAG-1 for the *Pristimantis orestes* species group, inferred with Maximum Likelihood. Bootstrap support is shown for nodes over 70%.

###
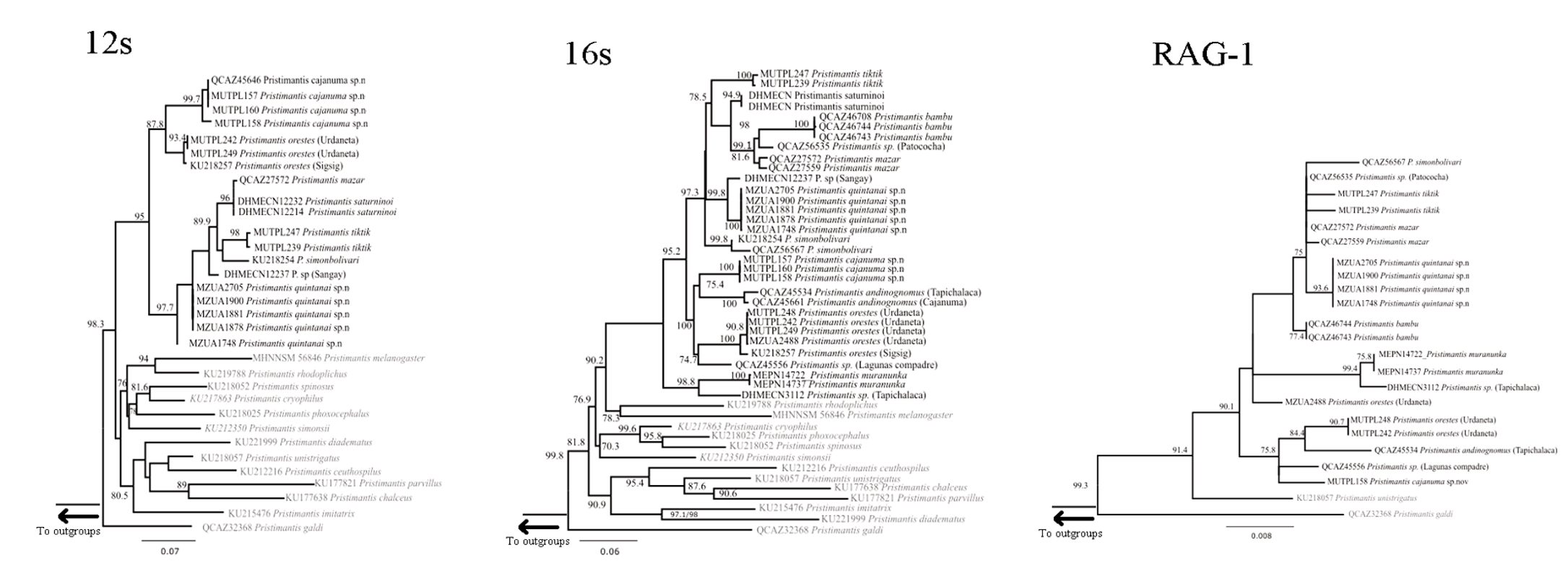

Supplement: Supplementary material 4 [file zookeys-864-111-s004.docx]
