# Supplementary material for: Genetic delimitation of Pristimantisorestes (Lynch, 1979) and P.saturninoi Brito et al., 2017 and description of two new terrestrial frogs from the Pristimantisorestes species group (Anura, Strabomantidae)
Source: Zookeys. 2019 Jul 18;864:111–46. doi: 10.3897/zookeys.864.35102 (PMC6658573; doi:10.3897/zookeys.864.35102)

### Supplementary material 5. Maximum Likelihood tree inference of the *Pristimantis orestes* species group. Bootstrap support values are shown for nodes over 70%. The bar coloration indicates the following: *P. cajanuma* sp. n (yellow), *P. orestes* (dark red), *P. saturninoi* (blue), and *P. quintanai* sp. n (green).

###
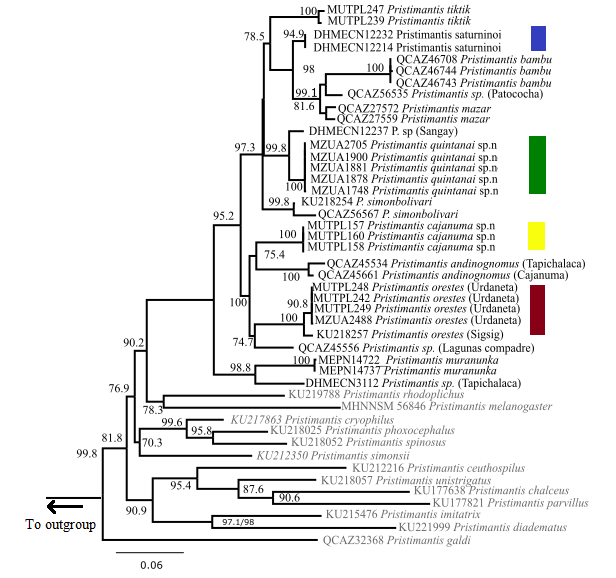

Supplement: Supplementary material 5 [file zookeys-864-111-s005.docx]
